# Discovery of C-3 Tethered 2-oxo-benzo[1,4]oxazines as Potent Antioxidants: Bio-Inspired Based Design, Synthesis, Biological Evaluation, Cytotoxic, and *in Silico* Molecular Docking Studies

**DOI:** 10.3389/fchem.2018.00056

**Published:** 2018-03-23

**Authors:** Vashundhra Sharma, Pradeep K. Jaiswal, Mukesh Saran, Dharmendra Kumar Yadav, Saloni, Manas Mathur, Ajit K. Swami, Sanjeev Misra, Mi-hyun Kim, Sandeep Chaudhary

**Affiliations:** ^1^Laboratory of Organic and Medicinal Chemistry, Department of Chemistry, Malaviya National Institute of Technology, Jaipur, India; ^2^Department of Advance Molecular Microbiology, Seminal Applied Sciences Pvt. Ltd., Jaipur, India; ^3^College of Pharmacy, Gachon University of Medicine and Science, Incheon, South Korea; ^4^Department of Biochemistry, All India Institute of Medical Sciences (AIIMS), Jodhpur, India

**Keywords:** 2-oxo-benzo[1,4]oxazines, antioxidant, DPPH, FRAP, ascorbic acid, BHT

## Abstract

The discovery of C-3 tethered 2-oxo-benzo[1,4]oxazines as potent antioxidants is disclosed. All the analogs **20a-20ab** have been synthesized via “on water” ultrasound-assisted irradiation conditions in excellent yields (upto 98%). All the compounds have been evaluated for their *in vitro* antioxidant activities using DPPH free radical scavenging assay as well as FRAP assay. The result showed promising antioxidant activities having IC_50_ values in the range of 4.74 ± 0.08 to 92.20 ± 1.54 μg/mL taking ascorbic acid (IC_50_ = 4.57 μg/mL) as standard reference. In this study, compounds **20b** and **20t**, the most active compound of the series, showed IC_50_ values of 6.89 ± 0.07 μg/mL and 4.74 ± 0.08 μg/mL, respectively in comparison with ascorbic acid. In addition, the detailed SAR study shows that electron-withdrawing group increases antioxidant activity and vice versa. Furthermore, in the FRAP assay, eight compounds (**20c**, **20j**, **20m**, **20n**, **20r**, **20u**, **20z**, and **20aa**) were found more potent than standard reference BHT (C_0.5FRAP_ = 546.0 ± 13.6 μM). The preliminary cytotoxic study reveals the non-toxic nature of active compounds **20b** and **20t** in non-cancerous 3T_3_ fibroblast cell lines in MTT assay up to 250 μg/mL concentration. The results were validated *via* carrying out *in silico* molecular docking studies of promising compounds **20a**, **20b**, and **20t** in comparison with standard reference. To the best of our knowledge, this is the first detailed study of C-3 tethered 2-oxo-benzo[1,4]oxazines as potential antioxidant agents.

## Introduction

“Antioxidant” are primarily reducing agents/compounds which refer to the activity of numerous vitamins, minerals and phytochemicals (such as vitamin E, vitamin C and glutathione etc.) by providing protection against the damage caused by reactive oxygen species (ROS) (Park and Pezzutto, 2002; Trombino et al., 2004; Govindarajan et al., 2005; Zhang et al., 2006). Antioxidants (either natural or synthetic) are molecules, which are capable of neutralizing free radicals as well as ROS by acting at several levels such as: prevention, interception, and repair (Lehtinen and Bonni, 2006; Khan et al., 2011; Bayoumi and Elsayed, 2012). Thus, the search for antioxidants has been stimulated due to their significant importance in human health (Balakin et al., 2004; Mitra et al., 2009). Moreover, it is known that ROS like superoxides (${\text{O}}_{2}^{2-}$), peroxyls (ROO^−^), hydroxyls (HO^−^), alkoxyls (RO^−^), nitric oxides (NO^−^), play a important role in disturbing metabolic pathways associated with several pathological conditions, such as cardiovascular diseases, metabolic disorders, and even carcinogenesis (Lai et al., 2001; Cheng et al., 2011). Therefore, the human body is capable to neutralize ROS by antioxidant defense mechanisms by eradicating an excess of ROS from the cell (Apel and Hirt, 2004; Zhang et al., 2010; Mittal et al., 2014). An imbalances between the detoxification of ROS with respect to their production leads to a phenomena known as “oxidative stress (OS)” which is correlated to several diseases such as stroke (Simao et al., 2015), myocardial infarction (Hassan et al., 2015), cancer (Aldawsari et al., 2016), Parkinson's disease (Wood-Kaczmar et al., 2006) and Alzheimer's disease (Nunomura et al., 2006). Therefore, the development of natural as well as synthetic antioxidants, which are able to scavenge ROS and keep cell integrity *via* prevention or reduction of the impact of OS on cells, is now currently an recognized area of research interest.

**Figure 1:**
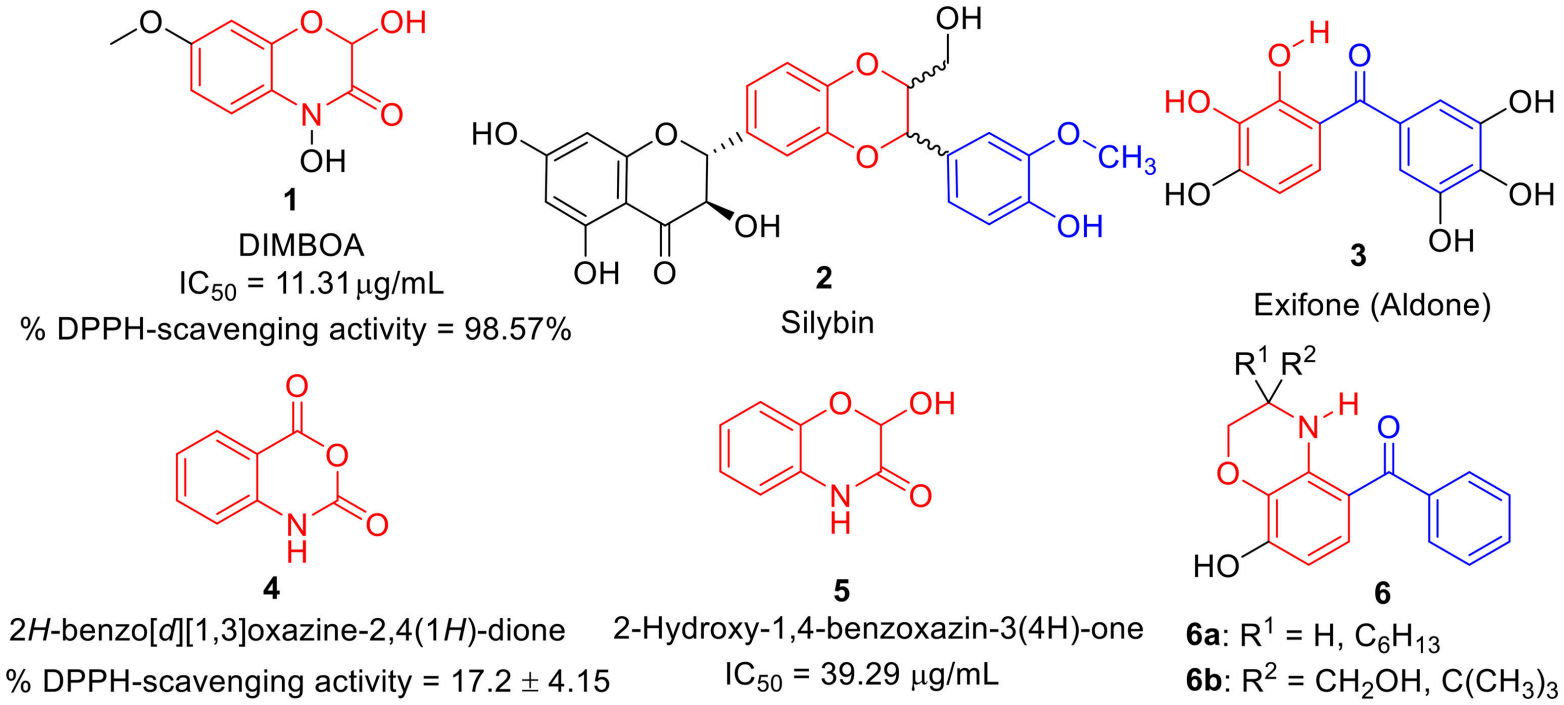
Structures of some natural as well as synthetic compounds **(1**-**6)** having antioxidant activity.

During the last decade, benzoxazines, benzodioxine, and its derivatives have emerged as a possible antioxidants (Largeron et al., 1999, 2001; Czompa et al., 2000; Sadiq et al., 2015). Several naturally occurring antioxidants (Abdel-lateif et al., 2016; Aziz and Karboune, 2016) such as dimboa **1** (Niemeyer, 2009; Adhikari et al., 2013; Glenska et al., 2015) and sylbin **2** (Kosina et al., 2002; Varga et al., 2006; Surai, 2015; Vavríkova et al., 2017) have been identified as promising antioxidant agents. Likewise, several synthetic molecules bearing benzoxazines as whole or as part in their structure, have also been identified as potential antioxidant agents such as exifone **3** (Largeron et al., 1995; Largeron and Fleury, 1998), isatoic anhydrides i.e., benzoxazine-2,4-diones **4** (Sáncheza et al., 2014), 2-hydroxy-1,4-benzoxazin-3(4H)-one **5** (Harput et al., 2011), and some analogs **6a-b** (Largeron et al., 1999) etc. as shown in Figure 1. This encourages us to synthesize non-naturally occurring benzo [1,4] oxazines analogs.

**Figure 2:**
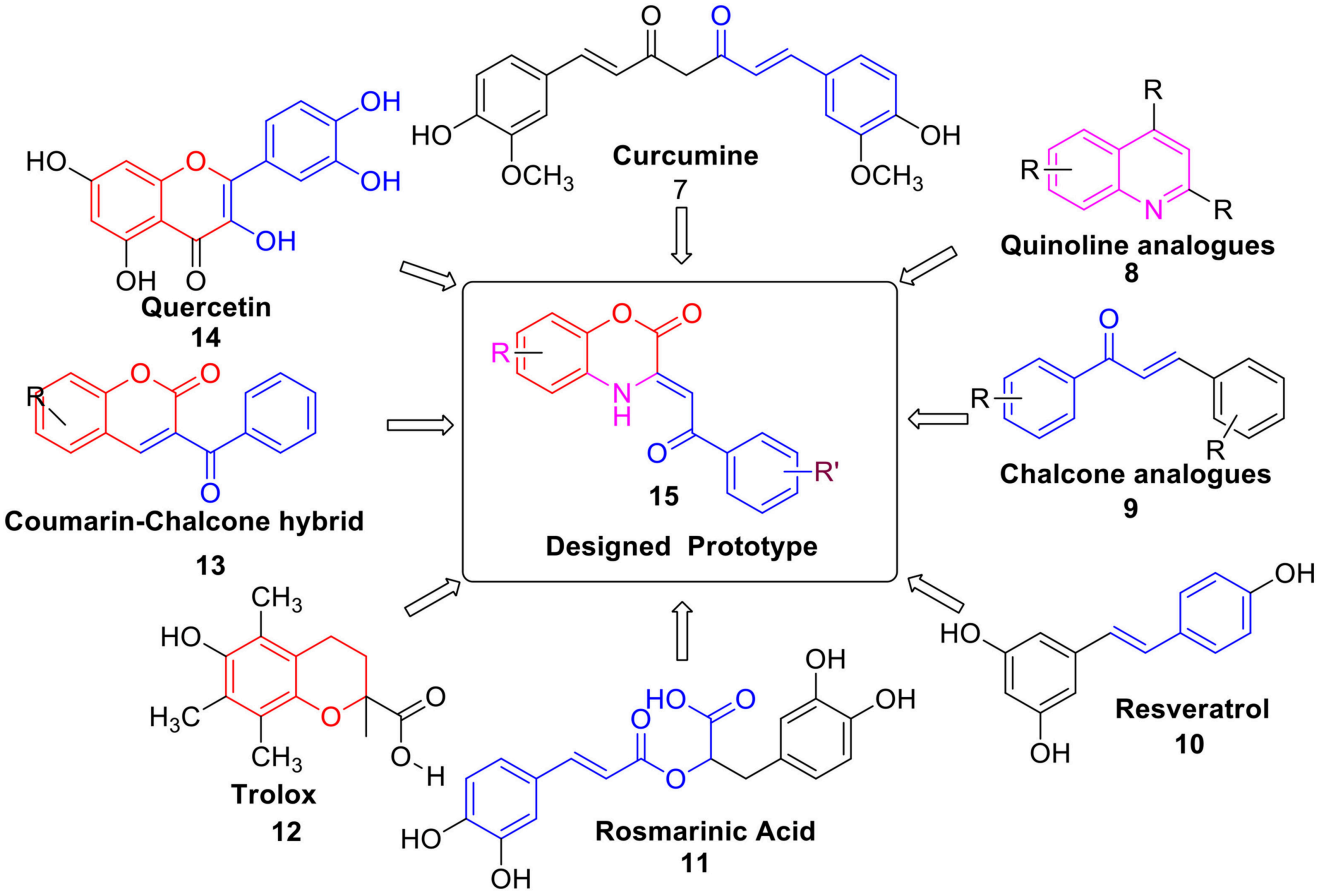
Design strategy for the target compound 2-oxo-benzo[1,4]oxazine **15** as an antioxidants.

Moreover, several natural products like Curcumine **7** (Barclay and Vinqvist, 2000), Quinolines **8** (Detsi et al., 2007; Savegnago et al., 2013; Oliveri et al., 2015), Chalcones **9** (Qian et al., 2011; Shakil et al., 2013; El Sayed Aly et al., 2014), Resveratrol **10** (Scartezzini and Speroni, 2000), Rosmarinic acid **11** (Fadel et al., 2011; Zhu et al., 2014), Trolox **12** (Hall et al., 2010a), Coumarins-chalcone hybrid **13** (PérezCruz et al., 2013; Mazzone et al., 2016), and Quercetin **14** (Kumar et al., 2007) etc. were also reported as antioxidants. However, due to several drawbacks such as poor solubility, less abundance and severe toxicity; their antioxidant properties were found to be relatively lower. Thus, there is still an urgent need to develop a potent antioxidant by designing a new scaffold *via* structural modification and incorporation of functional group present in these antioxidants. Hence, based on above fact, we have designed **prototype 15** i.e., C-3 tethered *2-oxo-benzo[1,4]oxazine*, incorporating similar sub-structural units assuming that the resulting structure will be a new class of potent antioxidant agent (Figure 2).

In the continuation toward the search of new class of antioxidants; we were interested to explore the designed prototype **15**. Therefore herein, we report the synthesis *via* our methodology (Jaiswal et al., 2017), antioxidant activity, and SAR of a series of C-3 tethered 2-oxo-benzo[1,4]oxazine analogs **20a**-**20ab**. Although compounds **20a-i**, **20l**, **20t-w**, **20y**, and **20aa-ab** have been earlier reported in the literature but were prepared by other routes (Iwanami et al., 1971; Mashevskaya et al., 2002; Gein et al., 2008; Xia, 2008; Xia et al., 2008; Stepanova et al., 2011, 2013; Maslivets and Maslivets, 2012). Moreover, their antioxidant activities are also not reported so far in the literature. To the best of our knowledge, the antioxidant activities of all the synthesized compounds **20a-20ab**, were evaluated for the first time using DPPH radical scavenging assay taking ascorbic acid as standard reference and FRAP assay using BHT as standard reference. In addition, the cytotoxic studies of active compounds were also performed. Moreover, we also report the validation of our results *via in silico* molecular docking studies of compounds **20a**, **20b** and **20t** in comparison with standard reference ascorbic acid.

## Materials and methods

### General experimental

All glass apparatus were oven dried prior to use. Melting points were taken in open capillaries on complab melting point apparatus and are presented uncorrected. Ultrasonic irradiation was performed in a Elmasonic S 30 (H) ultrasonic water bath cleaner and the reaction vessel was positioned in the maximum energy area in the cleaner and the removal or addition of water was used to control the temperature of the water bath. Infrared spectra were recorded on a Perkin-Elmer FT-IR Spectrum 2 spectrophotometer ^1^H NMR and ^13^C NMR spectra were recorded on ECS 400 MHz (JEOL) NMR spectrometer using CDCl_3_, CD_3_ODandCD_3_SOCD_3_ as solvent and tetramethylsilane as internal reference. Electrospray ionization mass spectrometry (ESI-MS) and HRMS were recorded on Xevo G2-S Q Tof (Waters, USA) Spectrometer. Column chromatography was performed over Merck silica gel (particle size: 60-120 Mesh) procured from Qualigens? (India), flash silica gel (particle size: 230-400 Mesh). All chemicals and reagents were obtained from Sigma Aldrich (USA), Merck (India) or Spectrochem (India) and were used without further purification.

### General procedure for the synthesis of functionalized diketo-acid 18a-h

Substituted acetophenone **16a-h** (2.00 mmol, 1eq.) were taken in toluene (50 ml) and NaH (2.20 mmol, 1.1 eq.) was added carefully. After stirring this reaction mixture at 0°C for 30 min dimethyl oxalate (2.20 mmol, 1.1 eq.) were added and reflux for 6 h. The progresses of the reaction were monitored by TLC using 9:1 Hexane/ethyl acetate as an eluent. After completion of reaction, the reaction mixture was quenched with distilled water and extracted with ethyl acetate (3 × 50 ml); then with distilled water (2 × 10 mL) followed by brine solution (2 × 20 mL). The organic layer was combined and dried over anhydrous Na_2_SO_4_ and the organic solvent was removed under reduced pressure to give the crude product. The crude products were purified by recrystalization using EtOAc/Hexane (v/v = 20:80), which afforded the pure desired diketo-ester **17a-h** in 78-92% yields. Compounds **17a-h** was used for next step without any further purification.

To a solution of **17a-h** (1.00 mmol, 1eq.) in MeOH:THF: H_2_O (10 ml, 7:2:1), added LiOH.H_2_O (1.20 mmol, 1.2eq) into the reaction mixture and stirred it for 4 h at room temperature. The progress of the reaction was monitored by TLC. After completion of the reaction, it was quenched with 3N HCl solution and extracted with ethyl acetate (3 × 30 mL); then with distilled water (2 × 10 mL) followed by brine solution (2 × 20 mL). The combined organic layer was dried over anhydrous Na_2_SO_4_ and evaporated under vacuum to afford the corresponding crude product. These crude products were further purified by recrystalization with EtOAc/Hexane, which afforded diketo-acids **18a-h** in excellent yields (up to 97%). Compounds **18a-h** were used for next step without any further purification.

### General procedure for the synthesis of functionalized (Z)-3-(2-oxo-2-phenylethylidene)-3,4-dihydro-2H-benzo[b][1,4]oxazin-2-one (20a-ab)

To a solution of the compound **18a-h** (0.20 mmol; 1eq.) in water (2.0 mL) was added compound **19a-f** (0.20 mmol; 1eq.) and the reaction mixture was irradiated under ultrasonic sonicator at 80°C temperature for about 75-90 min (depending upon the substrate employed). The progress of the reaction was checked by TLC using 9:1 Hexane/ethyl acetate as an eluent. After completion of reaction, the reaction mixture was extracted with ethyl acetate (3 × 50 ml); then with distilled water (2 × 10 mL) followed by brine solution (2 × 20 mL). The organic layers were combined and dried over anhydrous Na_2_SO_4_ and the organic solvent was removed under reduced pressure to give the crude product. The crude products were purified either by recrystalization using Hexane/EtOAc (v/v = 90:10) or by flash column chromatography method over silica gel using 7.5:2.5 to 9:1 Hexane/ethyl acetate as an eluent which afforded the pure desired (Z)-3-(2-oxo-2-phenylethylidene)-3,4-dihydro-2H-benzo[b][1,4]-oxazin-2-one **20a-ab** having good yields (80–98%).

### Characterization data of 2-oxo-benzo[1,4]oxazin-2-one (20a-ab)

#### (Z)-3-(2-oxo-2-phenylethylidene)-3,4-dihydro-2H-benzo[b][1,4]oxazin-2-one (20a)

Yellow solid; yield: 51.97 mg (98%), R_f_ (EtOAc/Hexane; 20:80) = 0.85; Purification of crude product was done by flash column chromatography method over silica gel using Hexane/ethyl acetate (9:1) as an eluent; m.p. 185–186°C; FT-IR (KBr, νmax/cm^−1^) 3434, 1754, 1614, 1594, 1270, 1113; ^1^H NMR (400 MHz, CDCl_3_) δ 8.00 (d, *J* = 7.4 Hz, 2H, Ar-H), 7.55–7.46 (m, 3H, Ar-H), 7.21–7.05 (m, 5H, C=CH, Ar-H); ^13^C NMR (100 MHz, CDCl_3_) δ 191.6 (C=O), 156.3 (O=C-O), 141.3 (C=CH), 139.1 (Ar-C), 138.3 (Ar-C), 132.8 (NH-C), 128.8 (Ar-C-NH), 127.7 (Ar-C), 126.0 (Ar-C), 124.0 (Ar-C), 123.8 (Ar-C), 117.2 (Ar-C), 116.0 (Ar-C), 94.7 (C=CH); HRMS (ESI) calcd. for C_16_H_11_NO_3_ [M+H]^+^: 266.0739; found 266.0734.

#### (Z)-3-[2-(4-methoxy-phenyl)-2-oxo-ethylidene]-3,4-dihydro-benzo [1, 4] oxazin-2-one (20b)

Yellowish solid; yield: 51.2 mg (88%), R_f_ (EtOAc/Hexane; 20:80) = 0.80; Purification of crude product was done by flash column chromatography method over silica gel using Hexane/ethyl acetate (8:2) as an eluent; m.p. 200–203°C; FT-IR (KBr, νmax/cm-1) 3435, 1756, 1602, 1112; ^1^H NMR (400 MHz, CDCl_3_) δ 7.99 (d, *J* = 8.8 Hz, 2H, Ar-H), 7.17 (t, J = 7.1 Hz, 2H, Ar-H), 7.09–7.05 (m, 2H, C=CH, Ar-H), 7.02–6.95 (m, 3H, Ar-H), 3.87 (s, 3H, O-CH_3_); ^13^C NMR (100 MHz, CDCl_3_) δ 190.5 (C=O), 163.5 (Ar-C-OCH_3_), 156.6 (O=C-O), 141.2 (Ar-C-N), 138.6 (Ar-C-O), 131.2 (C=CH), 130.0 (Ar-C), 125.9 (Ar-C), 124.0 (Ar-C), 123.7 (Ar-C), 117.2 (Ar-C), 115.8 (Ar-C), 114.0 (Ar-C), 94.7 (C=CH), 55.6 (O-CH_3_); HRMS (ESI) calcd. for C_17_H_13_NO_4_ [M+H]^+^: 296.0845; found 296.0849.

#### (Z)-6-chloro-3-(2-oxo-2-phenylethylidene)-3,4-dihydro-2H-benzo[b][1,4]oxazin-2-one (20c)

Yellowish solid; yield: 56.93 mg (95%), R_f_ (EtOAc/Hexane; 20:80) = 0.80; Purification of crude product was done by flash column chromatography method over silica gel using Hexane/ethyl acetate (8.5:1.5) as an eluent; m.p. 185–187°C; FT-IR (KBr, νmax/cm^−1^) 3434, 1761, 1555, 1622, 1174; ^1^H NMR (400 MHz, CDCl_3_) δ 8.00–7.98 (m, 2H, Ar-H), 7.58–7.55 (m, 1H, Ar-H), 7.50-7.47 (m, 2H, Ar-H), 7.13–7.03 (m, 4H, Ar-H, C=CH); ^13^C NMR (100 MHz, CDCl_3_) δ 191.8 (C=O), 155.8 (O=C-O), 139.8 (Ar-C-N), 138.4 (Ar-C-O), 138.1 (C=CH), 133.0 (Ar-C), 131.1 (Ar-C-Cl), 128.9 (Ar-C), 127.8 (Ar-C), 124.8 (Ar-C), 123.8 (Ar-C), 118.3 (Ar-C), 115.8 (Ar-C), 95.7 (C=CH); HRMS (ESI) calcd. for C_16_H_10_ClNO_3_ [M+2]^+^: 301.7085; found 301.7089.

#### (Z)-6-chloro-3-(2-(4-fluorophenyl)-2-oxoethylidene)-3,4-dihydro-2H-benzo[b][1,4]oxazin-2-one (20d)

Yellowish solid; yield: 59.08 mg (93%); R_f_ (EtOAc/Hexane; 20:80) = 0.80; Purification of crude product was done by flash column chromatography method over silica gel using Hexane/ethyl acetate (8.5:1.5) as an eluent; m.p. 155–157°C; FT-IR (KBr, νmax/cm^−1^) 3434,1754,1634,1601,1495, 1226,1160; ^1^H NMR (400 MHz, CDCl_3_) δ 8.06-8.02 (m, 2H, Ar-H), 7.20-7.13 (m, 4H, Ar-H), 7.08-7.04 (m, 2H, C=CH, Ar-H);^13^C NMR (100 MHz, CDCl_3_) δ 190.4 (C=O), 167.2 (Ar-C-F), 155.8 (O=C-O), 139.8 (Ar-C-N), 138.6 (Ar-C-O), 131.3 (Ar-C-Cl), 130.5 (C=CH), 130.4 (Ar-C), 124.8 (Ar-C), 123.9 (Ar-C), 118.4 (Ar-C), 116.2 (Ar-C), 115.9 (Ar-C), 115.8 (Ar-C), 95.4 (C=CH); HRMS (ESI) calcd. for C_16_H_9_ClFNO_3_ [M+H]^+^: 318.0255; found 318.0259.

#### (Z)-6-chloro-3-(2-(4-chlorophenyl)-2-oxoethylidene)-3,4-dihydro-2H-benzo[b][1,4]oxazin-2-one (20e)

Yellowish solid; yield: 63.9 mg (96%); R_f_ (EtOAc/Hexane; 20:80) = 0.90; Purification of crude product was done by flash column chromatography method over silica gel using Hexane/ethyl acetate (9:1) as an eluent; m.p. 182–185°C; FT-IR (KBr, νmax/cm^−1^) 3434, 1761, 1631, 1586, 1088; ^1^H NMR (400 MHz, CDCl_3_) δ 7.93 (d, *J* = 8.2 Hz, 2H, Ar-H), 7.45 (d, *J* = 8.1 Hz, 2H, Ar-H), 7.13–7.00 (m, 4H, C=CH, Ar-H); ^13^C NMR (100 MHz, CDCl_3_) δ 190.4 (C=O), 155.7 (O=C-O), 139.8 (Ar-C-Cl), 139.4 (Ar-C-N), 138.7 (Ar-C-O), 136.3 (C=CH), 131.2 (Ar-C), 129.2 (Ar-C), 129.1 (Ar-C), 124.6 (Ar-C), 124.0 (Ar-C), 118.3 (Ar-C), 115.9 (Ar-C), 95.3 (C=CH); HRMS (ESI) calcd. for C_16_H_9_Cl_2_NO_3_ [M+2]^+^: 334.9959; found 334.9956.

#### (Z)-6-chloro-3-(2-(2,4-dichlorophenyl)-2-oxoethylidene)- 3,4-dihydro-2H-benzo[b][1,4]oxazine-2-One (20f)

Yellowish solid; yield: 65.9 mg (89%); R_f_ (EtOAc/Hexane; 20:80) = 0.85; Purification of crude product was done by flash column chromatography method over silica gel using Hexane/ethyl acetate (9:1) as an eluent; m.p. 135–137°C; FT-IR (KBr, νmax/cm-1) 3432, 3075, 2923, 1626, 1583, 1105; ^1^H NMR (400 MHz, CDCl_3_) δ 7.54 (d, *J* = 8.4 Hz, 1H, Ar-H), 7.47 (d, *J* = 1.6 Hz, 1-H, Ar-H), 7.34 (dd, *J* = 1.6 Hz, 8.4 Hz, 1H, Ar-H), 7.17-7.09 (m, 3H, Ar-H), 6.82 (s, 1H, C=CH); ^13^C NMR (100 MHz, CDCl_3_) δ 192.1 (C=O), 155.4 (O=C-O), 139.9 (Ar-C-Cl), 138.4 (Ar-C-Cl), 137.7 (Ar-C-N), 137.2 (Ar-C-O), 132.7 (C=CH), 131.3 (Ar-C), 130.8 (Ar-C), 130.7 (Ar-C), 127.6 (Ar-C), 124.4 (Ar-C), 118.4 (Ar-C), 116.1 (Ar-C), 99.3 (C=CH); HRMS (ESI) calcd. for C_16_H_8_Cl_3_NO_3_ [M+2]^+^: 368.9570; found 368.9577.

#### (Z)-3-(2-(4-bromophenyl)-2-oxoethylidene)-6-chloro-3,4-dihydro-2H-benzo[b][1,4]oxazin-2-one (20g)

Yellowish solid; yield: 70.7 mg (93%); R_f_ (EtOAc/Hexane; 20:80) = 0.80; Purification of crude product was done by flash column chromatography method over silica gel using Hexane/ethyl acetate (9:1) as an eluent; m.p. 175–177°C; FT-IR (KBr, νmax/cm^−1^) 3436, 2924, 1755, 1632, 1583, 1007; ^1^H NMR (400 MHz, CDCl_3_) δ 7.85 (d, *J* = 7.9 Hz, 2H, Ar-H), 7.62 (d, *J* = 7.9 Hz, 2H, Ar-H), 7.13 – 7.00 (m, 4H, Ar-H, C=CH); ^13^C NMR (100 MHz, CDCl_3_) δ 190.5 (C=O), 155.6 (O=C-O), 139.8 (Ar-C-N), 138.8 (Ar-C-O), 136.8 (C=CH), 132.1 (Ar-C-Cl), 131.2 (Ar-C), 129.3 (Ar-C-Br), 128.1 (Ar-C), 124.6 (Ar-C), 124.0 (Ar-C), 118.3 (Ar-C), 115.9 (Ar-C), 95.2 (C=CH); HRMS (ESI) calcd. for C_16_H_9_BrClNO_3_ [M+2]^+^: 377.9454; found 377.9458.

#### (Z)-6-chloro-3-(2-oxo-2-(p-tolyl)ethylidene)-3,4-dihydro-2H-benzo[b][1,4]oxazin-2-one (20h)

Yellowish solid; yield: 56.5 mg (90%); R_f_ (EtOAc/Hexane; 20:80) = 0.90; Purification of crude product was done by flash column chromatography method over silica gel using Hexane/ethyl acetate (9.5:0.5) as an eluent; m.p. 160–162°C; FT-IR (KBr, νmax/cm-1) 3434, 2925, 1624, 1766, 1494, 1178; ^1^H NMR (400 MHz, CDCl_3_) δ 7.94–7.92 (m, 2H, Ar-H), 7.30 (d, *J* = 8.0 Hz, 2H, Ar-H), 7.14-7.04 (m, 4H, C=CH, Ar-H), 2.44 (s, 3H, Ar-CH_3_);^13^C NMR (100 MHz, CDCl_3_) δ 191.6 (C=O), 156.0 (O=C-O), 144.0 (Ar-C-CH_3_), 139.8 (Ar-C-N), 138.2 (Ar-C-O), 135.6 (C=CH), 131.2 (Ar-C), 129.7 (Ar-C), 128.0 (Ar-C), 125.0 (Ar-C), 123.7 (Ar-C), 118.3 (Ar-C), 115.8 (Ar-C), 95.9 (C=CH), 21.8 (-CH_3_); HRMS (ESI) calcd. for C_17_H_12_ClNO_3_ [M+H]^+^: 314.0506; found 314.0509.

#### (Z)-6-chloro-3-(2-(4-methoxyphenyl)-2-oxoethylidene)-3,4-dihydro-2H-benzo[b][1,4]oxazin-2-one (20i)

Yellowish solid; yield: 56.6 mg (86%); R_f_ (EtOAc/Hexane; 20:80) = 0.80; Purification of crude product was done by flash column chromatography method over silica gel using Hexane/ethyl acetate (9:1) as an eluent; m.p. 178-180°C; FT-IR (KBr, νmax/cm^−1^) 3434, 1764, 1628, 1594, 1018; ^1^H NMR (400 MHz, CDCl_3_) δ 7.99-7.97 (m, 2H, Ar-H), 7.10-7.06 (m, 2H, Ar-H), 7.02–6.99 (m, 2H, Ar-H), 6.97-6.94 (m, 2H, Ar-H, C=CH), 3.87 (s, 3H, OCH_3_); ^13^C NMR (100 MHz, CDCl_3_) δ 190.5 (C=O), 163.7 (Ar-C-OCH_3_), 156.1 (O=C-O), 139.6 (Ar-C-N), 137.8 (Ar-C-O), 131.1 (C=CH), 130.9 (Ar-C), 130.1 (Ar-C), 125.0 (Ar-C), 123.4 (Ar-C), 118.2 (Ar-C), 115.6 (Ar-C), 114.1 (Ar-C), 95.7 (C=CH), 55.6 (-OCH_3_); HRMS (ESI) calcd. for C_17_H_12_ClNO_4_ [M+2]^+^: 331.7345; found 331.7349.

#### (Z)-3-(2-(4-fluorophenyl)-2-oxoethylidene)-6-methyl-3,4-dihydro-2H-benzo[1,4]oxazin-2-one (20j)

Yellowish solid; yield: 53.7 mg (90%); R_f_ (EtOAc/Hexane; 20:80) = 0.85; Purification of crude product was done by flash column chromatography method over silica gel using Hexane/ethyl acetate (9.5:0.5) as an eluent; m.p. 145–147°C; FT-IR (KBr, νmax/cm^−1^) 3433, 2930, 1770, 1624, 1596, 1128; ^1^H NMR (400 MHz, CDCl_3_) δ 8.02 (dd, *J* = 5.6, 8.8 Hz, 2H, Ar-H), 7.17–7.07 (m, 3H, Ar-H), 6.98–6.90 (m, 3H, C=CH, Ar-H), 2.36 (s, 3H, CH_3_); ^13^C NMR (100 MHz, CDCl_3_) δ 190.0 (C=O), 166.9 (Ar-C-F), 156.4 (O=C-O), 139.3 (Ar-C-CH_3_), 136.1 (Ar-C-N), 134.7 (Ar-C-O), 130.3 (C=CH), 130.2 (Ar-C), 124.9 (Ar-C), 123.3 (Ar-C), 116.9 (Ar-C), 116.2 (Ar-C), 115.9 (Ar-C), 115.8 (Ar-C), 94.1 (C=CH), 21.1 (CH_3_); HRMS (ESI) calcd. for C_17_H_12_FNO_3_ [M+H]^+^: 298.0801; found 298.0807.

#### (Z)-3-[2-(2,4-dichloro-phenyl)-2-oxo-ethylidene]-6-methyl-3,4-dihydro-benzo[1,4]oxazin-2-one (20k)

Yellowish solid; yield: 65.4 mg (94%), R_f_ (EtOAc/Hexane; 20:80) = 0.80; Purification of crude product was done by recrystalization using Hexane/ethyl acetate; m.p. 142–145°C; FT-IR (KBr, νmax/cm^−1^) 3436, 2913, 1755, 1618, 1570, 1083; ^1^H NMR (400 MHz, CDCl_3_) δ 7.53 (d, *J* = 8.3 Hz, 1H, Ar-H), 7.45 (d, *J* = 2.0 Hz, 1H, Ar-H), 7.33–7.30 (m, 1H, Ar-H), 7.10–7.08 (m, 1H, Ar-H), 6.94–6.92 (m, 2H, Ar-H), 6.73 (s, 1H, C=CH), 2.36 (s, 3H, CH_3_); ^13^C NMR (100 MHz, CDCl_3_) δ 191.6 (C=O), 155.9 (O=C-O), 139.5 (Ar-C-CH_3_), 139.2 (Ar-C-Cl), 137.5 (Ar-C-Cl), 137.2 (Ar-C-N), 136.2 (Ar-C-O), 132.5 (C=CH), 130.7 (Ar-C), 130.6 (Ar-C), 127.4 (Ar-C), 125.4 (Ar-C), 123.0 (Ar-C), 117.0 (Ar-C), 116.4 (Ar-C), 98.0 (C=CH), 21.1 (CH_3_); HRMS (ESI) calcd. for C_17_H_11_Cl_2_NO_3_ [M+2]^+^: 349.0116; found 349.0112.

#### (Z)-3-[2-(4-methoxy-phenyl)-2-oxo-ethylidene]-6-methyl-3,4-dihydro-benzo[1,4]oxazin-2-one (20l)

Yellowish solid; yield: 57.7 mg (93%), R_f_ (EtOAc/Hexane; 20:80) = 0.75; Purification of crude product was done by flash column chromatography method over silica gel using Hexane/ethyl acetate (8:2) as an eluent; m.p. 180–182°C; FT-IR (KBr, νmax/cm^−1^) 3452, 1755, 1625, 1581; ^1^H NMR (400 MHz, CDCl_3_) δ 7.99 (d, *J* = 8.8 Hz, 2H, Ar-H), 7.06 (d, *J* = 8.0 Hz, 1H, Ar-H), 7.00 (s, 1H, Ar-H), 6.96 (d, *J* = 9.4 Hz, 2H, Ar-H), 6.88 – 6.86 (m, 2H, Ar-H, C=CH), 3.88 (s, 3H, OCH_3_), 2.34 (s, 3H, CH_3_); ^13^C NMR (100 MHz, CDCl_3_) δ 190.4 (C=O), 163.4 (Ar-C-OCH_3_), 156.8 (O=C-O), 139.3 (Ar-C-CH_3_), 138.7 (Ar-C-N), 136.0 (Ar-C-O), 131.3 (C=CH), 129.9 (Ar-C), 124.4 (Ar-C), 123.6 (Ar-C), 116.8 (Ar-C), 116.0 (Ar-C), 114.0 (Ar-C), 94.5 (C=CH), 55.6 (OCH_3_), 21.1 (CH_3_); HRMS (ESI) calcd. for C_18_H_15_NO_4_ [M+H]^+^: 310.1001; found 310.1009.

#### (Z)-8-bromo-6-methyl-3-(2-oxo-2-phenylethylidene)-3,4-dihydro-2H-benzo[b][1,4]oxazin-2-one (20m)

Yellowish solid; Yield: 65.7 mg (92%); Rf (EtOAc/Hexane; 20:80) = 0.80; Purification of crude product was done by flash column chromatography method over silica gel using Hexane/ethyl acetate (8.5:1.5) as an eluent; m.p. 190–192°C; FT-IR (KBr, νmax/cm^−1^) 3417, 1767, 1626, 1583, 1282, 1177; ^1^H NMR (400 MHz, CDCl_3_) δ 8.00 (d, *J* = 7.2 Hz, 2H, Ar-H), 7.58-7.47 (m, 3H, Ar-H), 7.13 (s, 1H, Ar-H), 7.07 (s, 1H, C=CH), 6.85 (s, 1H, Ar-H), 2.34 (s, 3H, CH_3_); ^13^C NMR (100 MHz, CDCl_3_) δ 191.7 (C=O), 155.7 (O=C-O), 138.6 (Ar-C-N), 138.1 (Ar-C-O), 136.9 (C=CH), 136.6 (Ar-C-CH_3_), 132.9 (Ar-C), 128.9 (Ar-C), 128.2 (Ar-C), 127.8 (Ar-C), 124.7 (Ar-C), 115.5 (Ar-C), 110.2 (Ar-C-Br), 95.2 (C=CH), 20.9 (CH_3_); HRMS (ESI) calcd. for C_17_H_12_BrNO_3_ [M+2]^+^: 359.0001; found 359.0008.

#### (Z)-8-bromo-3-(2-(4-fluorophenyl)-2-oxoethylidene)-6-methyl-3,4-dihydro-2H-benzo[b][1,4] oxazin-2-one (20n)

Yellowish solid; Yield: 67.6 mg (90 %); R_f_ (EtOAc/Hexane; 20:80) = 0.80; Purification of crude product was done by recrystalization using EtOAc/Hexane; m.p. 230–232°C; FT-IR (KBr, νmax/cm^−1^) 3417, 1771, 1631, 1285, 1159; ^1^H NMR (400 MHz, CDCl_3_) δ 8.03-7.99 (m, 2H, Ar-H), 7.17-7.13 (m, 3H, Ar-H), 6.99 (s, 1H, Ar-H), 6.84 (brs, 1H, C=CH), 2.34 (s, 3H, CH_3_); ^13^C NMR (100 MHz, CDCl_3_) δ 190.1 (C=O), 167.0 (Ar-C-F), 164.5 (Ar-C-F), 155.6 (O=C-O), 138.7 (Ar-C-N), 136.9 (Ar-C-O), 136.6 (C=CH), 134.5 (Ar-C-CH_3_), 134.4 (Ar-C), 130.4 (Ar-C), 130.3 (Ar-C), 128.3 (Ar-C), 124.6 (Ar-C-Br), 116.1 (Ar-C), 115.9 (Ar-C), 115.5 (Ar-C), 110.2 (Ar-C), 94.8 (C=CH), 20.9 (CH_3_); HRMS (ESI) calcd. for C_17_H_11_BrFNO_3_ [M+2]^+^: 376.9906; found 376.9909.

#### (Z)-8-bromo-3-(2-(2,4-dichlorophenyl)-2-oxoethylidene)-6-methyl-3,4-dihydro-2H-benzo [b][1,4]oxazin-2-one (20o)

Yellowish solid; Yield: 79.7 mg (94%); R_f_ (EtOAc/Hexane; 20:80) = 0.85; Purification of crude product was done by recrystalization using Hexane/ethyl acetate; m.p. 224–226°C; FT-IR (KBr, νmax/cm^−1^) 3417, 1763, 1626, 1561, 1294, 1127; ^1^H NMR (400 MHz, CDCl_3_) δ 7.54 (d, *J* = 8.4 Hz, 1H, Ar-H), 7.47 (s, 1H, Ar-H), 7.33 (d, *J* = 8.0 Hz, 1H, Ar-H), 7.18 (s, 1H, Ar-H), 6.88 (s, 1H, C=CH), 6.79 (s, 1H, Ar-H), 2.35 (s, 3H, CH_3_); ^13^C NMR (100 MHz, CDCl_3_) δ 191.9 (C=O), 155.2 (O=C-O), 138.7 (Ar-C-Cl), 137.5 (Ar-C-N), 137.3(Ar-C-O), 137.0 (C=CH), 136.8 (Ar-C-CH_3_), 132.7 (Ar-C-Cl), 130.8 (Ar-C), 130.7 (Ar-C), 128.8 (Ar-C), 127.5 (Ar-C), 124.3 (Ar-C), 115.7 (Ar-C), 110.3 (Ar-C), 98.8 (C=CH), 20.9 (CH_3_); HRMS (ESI) calcd. for C_17_H_10_BrCl_2_NO_3_ [M+2]^+^: 426.9221; found 426.9227.

#### (Z)-8-bromo-3-(2-(4-bromophenyl)-2-oxoethylidene)-6-methyl-3,4-dihydro-2H-benzo[b] [1,4]oxazin-2-one (20p)

Yellowish solid; Yield: 77.6 mg (89%); R_f_ (EtOAc/Hexane; 20:80) = 0.90; Purification of crude product was done by flash column chromatography method over silica gel using Hexane/ethyl acetate (9.5:0.5) as an eluent; m.p. 259–260°C; FT-IR (KBr, νmax/cm^−1^) 3417, 1768, 1629, 1562, 1283, 1138; ^1^H NMR (400 MHz, CDCl_3_) δ 7.86 (d, *J* = 8.8 Hz, 2H, Ar-H), 7.62 (d, *J* = 8.4 Hz, 2H, Ar-H), 7.16 (s, 1H, Ar-H), 7.00 (s, 1H, Ar-H), 6.86 (s, 1H, C=CH), 2.35 (s, 3H, CH_3_); ^13^C NMR (100 MHz, CDCl_3_) δ 190.4 (C=O), 155.6 (O=C-O), 139.0 (Ar-C-N), 137.0 (Ar-C-O), 136.9 (C=CH), 136.7 (Ar-C-CH_3_), 132.2 (Ar-C), 129.3 (Ar-C), 128.5 (Ar-C), 128.1 (Ar-C), 124.5 (Ar-C), 115.6 (Ar-C), 110.3 (Ar-C), 94.8 (C=CH), 20.9 (CH_3_); HRMS (ESI) calcd. for C_17_H_11_Br_2_NO_3_ [M+2]^+^: 436.9106; found 436.9100.

#### (Z)-8-bromo-6-methyl-3-(2-oxo-2-(p-tolyl)ethylidene)-3,4-dihydro-2H-benzo[b][1,4]oxazin-2-one (20q)

Yellowish solid; Yield: 70.7 mg (95%); R_f_ (EtOAc/Hexane; 20:80) = 0.90; Purification of crude product was done by flash column chromatography method over silica gel using Hexane/ethyl acetate (8.5:1.5) as an eluent; m.p. 219–220°C; FT-IR (KBr, νmax/cm^−1^) 3417, 1764, 1630, 1602, 1281, 1182; ^1^H NMR (400 MHz, CDCl_3_) δ 7.86 (d, *J* = 8.4 Hz, 2H, Ar-H), 7.26–7.24 (m, 2H, Ar-H), 7.08 (s, 1H, Ar-H), 7.00 (s, 1H, Ar-H), 6.80 (s, 1H, C=CH), 2.39 (s, 3H, CH_3_), 2.30 (s, 3H, CH_3_); ^13^C NMR (100 MHz, CDCl_3_) δ 191.4 (C=O), 155.8 (O=C-O), 143.9 (Ar-C-CH_3_), 138.3 (Ar-C-N), 136.8 (Ar-C-O), 136.5 (C=CH), 135.6 (Ar-C-CH_3_), 129.6 (Ar-C), 128.0 (Ar-C), 127.9 (Ar-C), 124.8 (Ar-C), 115.4 (Ar-C), 110.1 (Ar-C), 95.3 (C=CH), 21.8 (CH_3_), 20.9 (CH_3_); HRMS (ESI) calcd. for C_18_H_14_BrNO_3_ [M+2]^+^: 373.0157; found 373.0152.

#### (Z)-6-methyl-3-(2-(4-nitrophenyl)-2-oxoethylidene)-3,4-dihydro-2H-benzo[1,4]oxazin-2-one (20r)

Yellowish solid; yield: 57.2 mg (82%), R_f_ (EtOAc/Hexane; 20:80) = 0.75; Purification of crude product was done by recrystalization using Hexane/ethyl acetate; m.p. 211–213°C; FT-IR (KBr, νmax/cm^−1^) 3446, 3072, 1758, 1621, 1515, 1183; ^1^H NMR (400 MHz, CDCl_3_) δ 8.32 (d, *J* = 8.4 Hz, 2H, Ar-H), 8.13 (d, *J* = 8.4 Hz, 2H, Ar-H), 7.12 (d, *J* = 7.6 Hz, 1H, Ar-H), 7.01–6.96 (m, 3H, Ar-H, C=CH), 2.37 (s, 3H, CH_3_); ^13^C NMR (100 MHz, CDCl_3_) δ 188.8 (C=O), 155.9 (O=C-O), 149.9 (Ar-C-NO_2_), 143.3 (C=CH), 140.5 (Ar-C-N), 139.7 (Ar-C-O), 136.4 (Ar-C), 128.6 (Ar-C), 125.8 (Ar-C), 124.0 (Ar-C), 123.9 (Ar-C), 117.2 (Ar-C), 116.7 (Ar-C), 94.1 (C=CH), 21.1 (CH_3_); HRMS (ESI) calcd. for C_17_H_12_N_2_O_5_ [M+H]^+^: 325.0746; found 325.0748.

#### (Z)-3-(2-(4-nitrophenyl)-2-oxoethylidene)-3,4-dihydro-2H-benzo[b][1,4]oxazin-2-one (20s)

Yellowish solid; yield: 52.2 mg (84%); R_f_ (EtOAc/Hexane; 20:80) = 0.75; Purification of crude product was done by recrystalization using Hexane/ethyl acetate; m.p. 207–209°C; FT-IR (KBr, νmax/cm^−1^) 3448, 3069, 1758, 1621, 1515; 1453; ^1^H NMR (400 MHz, CDCl_3_) δ 8.32 (d, *J* = 8.8 Hz, 2H, Ar-H), 8.15 (d, *J* = 7.2 Hz, 2H, Ar-H), 7.26 – 7.17 (m, 4H, Ar-H), 7.04 (s, 1H, C=CH); ^13^C NMR (100 MHz, CDCl_3_) δ 188.9 (C=O), 155.8 (O=C-O), 150.1 (Ar-C-NO_2_), 143.3 (Ar-C-N), 141.7 (C=CH), 140.5 (Ar-C-O), 128.7 (Ar-C), 126.2 (Ar-C), 125.1 (Ar-C), 124.1 (Ar-C), 123.4 (Ar-C), 117.5 (Ar-C), 116.6 (Ar-C), 94.3 (C=CH); HRMS (ESI) calcd. for C_16_H_10_N_2_O_5_ [M+H]^+^: 311.0590; found 311.0596.

#### (Z)-6-nitro-3-(2-oxo-2-phenylethylidene)-3,4-dihydro-2H-benzo[b][1,4]oxazin-2-one (20t)

Yellowish solid; yield: 55.21 mg (89%); R_f_ (EtOAc/Hexane; 20:80) = 0.75; Purification of crude product was done by flash column chromatography method over silica gel using Hexane/ethyl acetate (7.5:2.5) as an eluent; m.p. 198–200°C; FT-IR (KBr, νmax/cm^−1^) 3436, 2928, 1762, 1625, 1581, 1142; ^1^H NMR (400 MHz, CDCl_3_) δ 8.03-7.96 (m, 4H, Ar-H), 7.61-7.49 (m, 3H, Ar-H), 7.31 (d, *J* = 8.8 Hz, 1H, Ar-H), 7.15 (s, 1H, C=CH); ^13^C NMR (100 MHz, CDCl_3_) δ 192.1 (C=O), 155.1 (O=C-O), 145.2 (Ar-C-NO_2_), 144.9 (C=CH), 137.7 (Ar-C-N), 137.6 (Ar-C-O), 133.4 (Ar-C), 128.9 (Ar-C), 127.9 (Ar-C), 124.7 (Ar-C), 118.9 (Ar-C), 118.0 (Ar-C), 111.5 (Ar-C), 96.9 (C=CH); HRMS (ESI) calcd. for C_16_H_10_N_2_O_5_ [M+H]^+^: 311.0590; found 311.0593.

#### (Z)-3-(2-(4-fluorophenyl)-2-oxoethylidene)-6-nitro-3,4-dihydro-2H-benzo[b][1,4]oxazin-2-one (20u)

Yellowish solid; yield: 61.71 mg (94%); R_f_ (EtOAc/Hexane; 20:80) = 0.75; Purification of crude product was done by flash column chromatography method over silica gel using Hexane/ethyl acetate (8:2) as an eluent; m.p. > 250°C; FT-IR (KBr, νmax/cm^−1^) 3435, 3107, 1759,1622, 1594, 1156; ^1^H NMR (400 MHz, DMSO-*d*_6_) δ 8.73 (s, 1H, Ar-H), 8.11 (d, *J* = 5.2 Hz, 2H, Ar-H), 7.92 (d, *J* = 6.4 Hz, 1H, Ar-H), 7.44 – 7.36 (m, 3H, Ar-H), 6.92 (s, 1H, C=CH); ^13^C NMR (100 MHz) δ 188.7 (C=O), 166.6, (Ar-C-F), 156.0 (O=C-O), 145.9 (Ar-C-NO_2_), 144.6 (C=CH), 139.5 (Ar-C-N), 135.1 (Ar-C-O), 130.9 (Ar-C), 125.8 (Ar-C), 118.9 (Ar-C), 117.7 (Ar-C), 116.6 (Ar-C), 116.4 (Ar-C), 113.0 (Ar-C), 94.5 (C=CH); HRMS (ESI) calcd. for C_16_H_9_FN_2_O_5_ [M+H]^+^: 329.0495; found 329.0490.

#### (Z)-3-(2-(4-methoxyphenyl)-2-oxoethylidene)-6-nitro-3,4-dihydro-2H-benzo[b][1,4]oxazin-2-one (20v)

Yellowish solid; yield: 58.51 mg (86%); R_f_ (EtOAc/Hexane; 20:80) = 0.75; Purification of crude product was done by flash column chromatography method over silica gel using Hexane/ethyl acetate (7:3) as an eluent; m.p. 195–197°C; FT-IR (KBr, νmax/cm^−1^) 3435, 2926, 1599, 1758, 1633, 1594; ^1^H NMR (400 MHz, CDCl_3_) δ 8.02–7.93 (m, 4H, Ar-H), 7.29 (d, *J* = 8.9 Hz, 1H, Ar-H), 7.10 (s, 1H, C=CH), 6.98 (d, *J* = 8.7 Hz, 2H, Ar-H), 3.89 (s, 3H, OCH_3_); ^13^C NMR (100 MHz, CDCl_3_) δ 190.7 (C=O), 164.0 (Ar-C-OCH_3_), 155.3 (O=C-O), 145.2 (Ar-C-NO_2_), 144.9 (Ar-C-N), 137.0 (Ar-C-O), 130.7 (C=CH), 130.3 (Ar-C), 124.9 (Ar-C), 118.6 (Ar-C), 117.8 (Ar-C), 114.2 (Ar-C), 111.2 (Ar-C), 97.0 (C=CH), 55.7 (OCH_3_); HRMS (ESI) calcd. for C_17_H_12_N_2_O_6_ [M+H]^+^: 341.0695; found 341.0692.

#### (Z)-7-nitro-3-(2-oxo-2-phenylethylidene)-3,4-dihydro-2H-benzo[b][1,4]oxazin-2-one (20w)

Yellowish solid; yield: 55.22 mg (89%); R_f_ (EtOAc/Hexane; 20:80) = 0.70; Purification of crude product was done by flash column chromatography method over silica gel using Hexane/ethyl acetate (7.5:2.5) as an eluent; m.p. 240–242°C; FT-IR (KBr, νmax/cm-1) 3436, 1763, 1622, 1596, 1268; ^1^H NMR (400 MHz, CDCl_3_) δ 8.06 – 8.00 (m, 4H, Ar-H), 7.83–7.81 (m, 1H, Ar-H), 7.62 (t, J = 7.3 Hz, 1H, Ar-H), 7.54 (t, J = 7.5 Hz, 2H, Ar-H), 6.99 (s, 1H, C=CH); ^13^C NMR (100 MHz, CDCl_3_) δ 190.7 (C=O), 156.1 (O=C-O), 142.2 (Ar-C-N), 141.1 (Ar-C-O), 139.2 (Ar-C-NO_2_), 138.3 (C=CH), 133.6 (Ar-C), 131.3 (Ar-C), 129.6 (Ar-C), 128.0 (Ar-C), 121.4 (Ar-C), 117.4 (Ar-C), 112.6 (Ar-C), 96.0 (C=CH); HRMS (ESI) calcd. for C_16_H_10_N_2_O_5_ [M+H]^+^: 311.0590; found 311.0595.

#### (Z)-3-(2-(4-fluorophenyl)-2-oxoethylidene)-7-nitro-3,4-dihydro-2H-benzo[b][1,4]oxazin-2-one (20x)

Yellowish solid; yield: 52.7 mg (80%); R_f_ (EtOAc/Hexane; 20:80) = 0.70; Purification of crude product was done by recrystalization using Hexane/ethyl acetate; m.p. 235–237°C; FT-IR (KBr, νmax/cm-1) 3411, 3090, 1773, 1626, 1516, 1473; ^1^H NMR (400 MHz, DMSO-*d*_6_) δ 8.18 - 8.08 (m, 4H, Ar-H), 7.88 – 7.86 (m, 1H, Ar-H), 7.42–7.38 (m, 2H, Ar-H), 7.02 (s, 1H, C=CH); ^13^C NMR (100 MHz, DMSO-*d*_6_) 188.7 (C=O), 163.7 (Ar-C-F), 155.5 (O=C-O), 141.8 (Ar-C-N), 140.6 (Ar-C-O), 138.7 (Ar-C-NO_2_), 134.4 (C=CH), 130.7 (Ar-C), 130.6 (Ar-C), 130.5 (Ar-C), 120.9 (Ar-C), 116.9 (Ar-C), 116.2 (Ar-C), 115.9 (Ar-C), 112.0 (Ar-C), 95.3 (C=CH); HRMS (ESI) calcd. for C_16_H_9_FN_2_O_5_ [M+H]^+^: 329.0495; found 329.0499.

#### (Z)-3-(2-(4-chlorophenyl)-2-oxoethylidene)-7-nitro-3,4-dihydro-2H-benzo[b][1,4]oxazin-2-one (20y)

Yellowish solid; yield: 59.7 mg (87%); R_f_ (EtOAc/Hexane; 20:80) = 0.70; Purification of crude product was done by flash column chromatography method over silica gel using Hexane/ethyl acetate (7.5:2.5) as an eluent; m.p. 205–207°C; FT-IR (KBr, νmax/cm-1) 3432, 2925, 2860, 1633, 1525, 1776, 1075; ^1^H NMR (400 MHz, DMSO-*d*_6_) δ 8.08 (d, *J* = 8.4 Hz, 4H, Ar-H), 7.89-7.87 (m, 1H, Ar-H), 7.63 (d, J = 8.4 Hz, 2H, Ar-H), 7.01 (s, 1H, C=CH); ^13^C NMR (100 MHz, DMSO-*d*_6_) δ 188.9 (C=O), 155.4 (O=C-O), 141.9 (Ar-C-N), 140.7 (Ar-C-Cl), 138.9 (Ar-C-O), 136.5 (Ar-C-NO_2_), 130.7 (C=CH), 129.8 (Ar-C), 129.5 (Ar-C), 129.2 (Ar-C), 120.9 (Ar-C), 117.0 (Ar-C), 112.1 (Ar-C), 95.2 (C=CH); HRMS (ESI) calcd. for C_16_H_9_ClN_2_O_5_ [M+H]^+^: 345.0200; found 345.0207.

#### (Z)-3-(2-(2,4-dichlorophenyl)-2-oxoethylidene)-7-nitro-3,4-dihydro-2H-benzo[b][1,4]oxazin-2-one (20z)

Yellowish solid; yield: 64.6 mg (85%); R_f_ (EtOAc/Hexane; 20:80) = 0.70; Purification of crude product was done by recrystalization using Hexane/ethyl acetate; m.p. 208–210°C; FT-IR (KBr, νmax/cm^−1^) 3433, 3088, 1770, 1620, 1522, 1470, 1072; ^1^H NMR (400 MHz, DMSO-*d*_6_) δ 12.39 (s, 1H, NH), 8.09–8.08 (m, 2H, Ar-H), 7.95–7.93 (s, 1H, Ar-H), 7.78–7.58 (m, 3H, Ar-H), 6.62 (s, 1H, C=CH); ^13^C NMR (100 MHz, DMSO-*d*_6_) δ 190.3 (C=O), 155.3 (O=C-O), 142.2 (Ar-C-N), 140.8 (Ar-C-Cl), 138.5 (Ar-C-O), 137.7 (Ar-C-NO_2_), 136.2 (C=CH), 131.2 (Ar-C-Cl), 131.0 (Ar-C), 130.5 (Ar-C), 130.1 (Ar-C), 127.9 (Ar-C), 120.8 (Ar-C), 117.3 (Ar-C), 112.1 (Ar-C), 98.8 (C=CH); HRMS (ESI) calcd. for C_16_H_8_Cl_2_N_2_O_5_ [M+H]^+^: 378.9810; found 378.9818.

#### (Z)-3-(2-(4-bromophenyl)-2-oxoethylidene)-7-nitro-3,4-dihydro-2H-benzo[b][1,4]oxazin-2-one(20aa)

Yellowish solid; yield: 67.7 mg (87%); R_f_ (EtOAc/Hexane; 20:80) = 0.70; Purification of crude product was done by flash column chromatography method over silica gel using Hexane/ethyl acetate (7.5:2.5) as an eluent; m.p. 230–232°C; FT-IR (KBr, νmax/cm^−1^) 3435, 3093, 1769, 1619, 1521; ^1^H NMR (400 MHz, DMSO-*d*_6_) δ 8.09-7.96 (m, 4H, Ar-H), 7.83-7.44 (m, 3H, Ar-H), 7.01(d, *J* = 4.8 Hz, 1H, C=CH); ^13^C NMR (100 MHz, DMSO-*d*_6_) δ 188.9 (C=O), 154.7 (O=C-O), 141.8 (Ar-C-N), 140.1 (Ar-C-O), 137.9 (C=CH), 136.6 (Ar-C-NO_2_), 131.5 (Ar-C), 130.0 (Ar-C), 129.1 (Ar-C-Br), 126.6 (Ar-C), 120.7 (Ar-C), 116.5 (Ar-C), 111.4 (Ar-C), 95.7 (C=CH); HRMS (ESI) calcd. for C_16_H_9_BrN_2_O_5_ [M+2]^+^: 389.9695; found 389.9691.

#### (Z)-3-(2-(4-methoxyphenyl)-2-oxoethylidene)-7-nitro-3,4-dihydro-2H-benzo[b][1,4]oxazin-2-one (20ab)

Yellowish solid; yield: 55.4 mg (81%); R_f_ (EtOAc/Hexane; 20:80) = 0.70; Purification of crude product was done by flash column chromatography method over silica gel using Hexane/ethyl acetate (7:3) as an eluent; m.p. 218–220°C; FT-IR (KBr, νmax/cm^−1^) 3437, 2927, 2854, 1632, 1517; ^1^H NMR (400 MHz, DMSO-*d*_6_) δ 8.07-8.02 (m, 4H, Ar-H), 7.73 (d, *J* = 10.8 Hz, 1H, Ar-H), 7.09 (d, *J* = 8.8 Hz, 2H, Ar-H), 7.02 (s, 1H, C=CH), 3.89 (s, 3H, OCH_3_); ^13^C NMR (100 MHz, DMSO-*d*_6_) δ 189.1 (C=O), 163.4 (Ar-C-OCH_3_), 155.8 (O=C-O), 141.7 (Ar-C-N), 140.6 (Ar-C-O), 138.1 (C=CH), 131.1 (Ar-C-NO_2_), 130.7 (Ar-C), 130.1 (Ar-C), 121.2 (Ar-C), 116.8 (Ar-C), 114.5 (Ar-C), 112.2 (Ar-C), 95.9 (C=CH), 55.8 (OCH_3_); HRMS (ESI) calcd. for C_17_H_12_N_2_O_6_ [M+H]^+^: 341.0695; found 341.0699.

### Pharmacological assay descriptions

#### DPPH radical scavenging antioxidant assay

In DPPH radical scavenging method the plant extract (0.75 mL) at different concentrations ranging from 10 to 100 μg mL^−1^ was mixed with 1.5 mL of a DPPH methanolic solution (20 mg L^−1^). Pure methanol was taken as control and ascorbic acid (vitamin C), vitamins A and E were used as a reference compounds. The absorbance was measured at 517 nm after 20 min of reaction. The % of DPPH decolouration of the sample was calculated according to the formula (Sharma and Bhat, 2009).

Decolouration % = [1−(Abs SAMPLE/Abs CONTROL)]×100

The decolouration was plotted against the sample extract concentration and a logarithmic regression curve was established in order to calculate the IC_50_. The results are expressed as antiradical efficiency (AE), which is 1000-fold inverse of the IC_50_ value AE=1000/ IC_50_.

#### Ferric reducing antioxidant power (FRAP) assay

The FRAP reagent was prepared by the addition of freshly prepared 20.0 mM FeCl_3_.6H_2_O solution, 10.0 mM of ferric-tripyridyltriazine (TPTZ) solution and 300 mM sodium acetate buffer (pH 3.6) in a ratio of 1:1:10 (v/v/v). After that, Sample (our synthesized 2-oxo-2-phenylethylidenes-linked 2-oxo-benzo[1,4]oxazines **20a-ab)** was added to 3 ml of freshly prepared FRAP reagent and this reaction mixture was incubated at 37°C temperature for 30 min. and the absorbance was measured at 593 nm. It is also noted that, a freshly prepared solution of FeSO_4_ was used for calibration of standard curve. The FRAP antioxidant capability were evaluated in terms of C_0.5_FRAP (the antioxidant capability of samples related to their concentration, which is equivalent to that of FeSO_4_ at 0.5 mmol/L) (Benzie and Strain, 1996).

#### Cell toxicity assay

Cell toxicity of active C-3 tethered 2-oxo-benzo[1,4]oxazine analogs were accessed using 3T_3_ fibroblast cell lines in MTT assay *via* the reported protocol of Danihelová et al. (2013) [For details, see supporting information].

#### *In silico* molecular docking studies

Molecular modeling studies of C-3 tethered 2-oxo-benzo [1, 4] oxazine derivatives **20a**-**ab** were carried out using molecular modeling software Sybyl-X 2.0, (Tripos International, St. Louis, Missouri, 63144, USA). Drawing of structures and simple geometry optimization were performed with Chem Bio-Office suite Ultra v12.0 (2012) (Cambridge Soft Corp., UK). Docking of all compounds was carried out on the human antioxidant enzyme in complex (PDB ID: 3MNG) (Hall et al., 2010b; Bayoumi et al., 2012; Yapati et al., 2016). The Surflexdoc module in Sybyl was used to construct a 3D model of the structures.

To find the possible bioactive conformations of C-3 tethered 2-oxo-benzo[1,4]oxazine derivatives, molecular modeling studies were performed using the Sybyl X 2.0 interfaced for the synthesized compounds, which exhibited promising and lower antioxidant activity *in vitro* to find the preferred binding conformations in the receptor. The starting coordinates of the human antioxidant enzyme in complex with the competitive inhibitor DTT (PDB: 3MNG) were taken from the Protein Data Bank (http://www.rcsb.org/pdb). Program automatically docks ligand into binding pocket of a target protein by using protomol-based algorithm and empirically produced scoring function. The protomol is very important and necessary factor for docking algorithm and works as a computational representation of proposed ligand that interacts into binding site. Surflex-Dock's scoring function have several factors that play an important role in the ligand-receptor interaction, in terms of hydrophobic, polar, repulsive, entropic and solvation, and it is a worldwide well-established and recognized method. The most standard docking protocols have ligand flexibility into the docking process, while counts the protein as a rigid structure. Present molecular docking study involves the several steps *viz*., import of protein structure into Surflex and addition of hydrogen atoms; generation of protomol using a ligand-based strategy. During second step, two parameters first called *protomol*^_^*bloat*, which determines how far the site should extend from a potential ligand; and another called *protomol*^_^*threshold*, which determines deepness of the atomic probes, used to define the protomol penetration into the protein) were specified to form the appropriate binding pocket. Therefore, protomol^_^bloat and protomol^_^threshold was set to 0 and 0.50, respectively. In reasonable binding pocket, all the compounds were docked into the binding pocket and 20 possible active docking conformations with different scores were obtained for each compound. During the docking process, all of the other parameters were assigned their default values.

## Results and discussion

### Chemistry

**Scheme 1:**
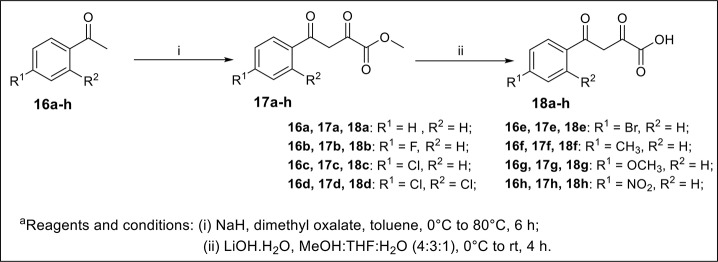
Synthesis of starting substrate functionalized diketo-acid **(18a-h)**.

**Scheme 2:**
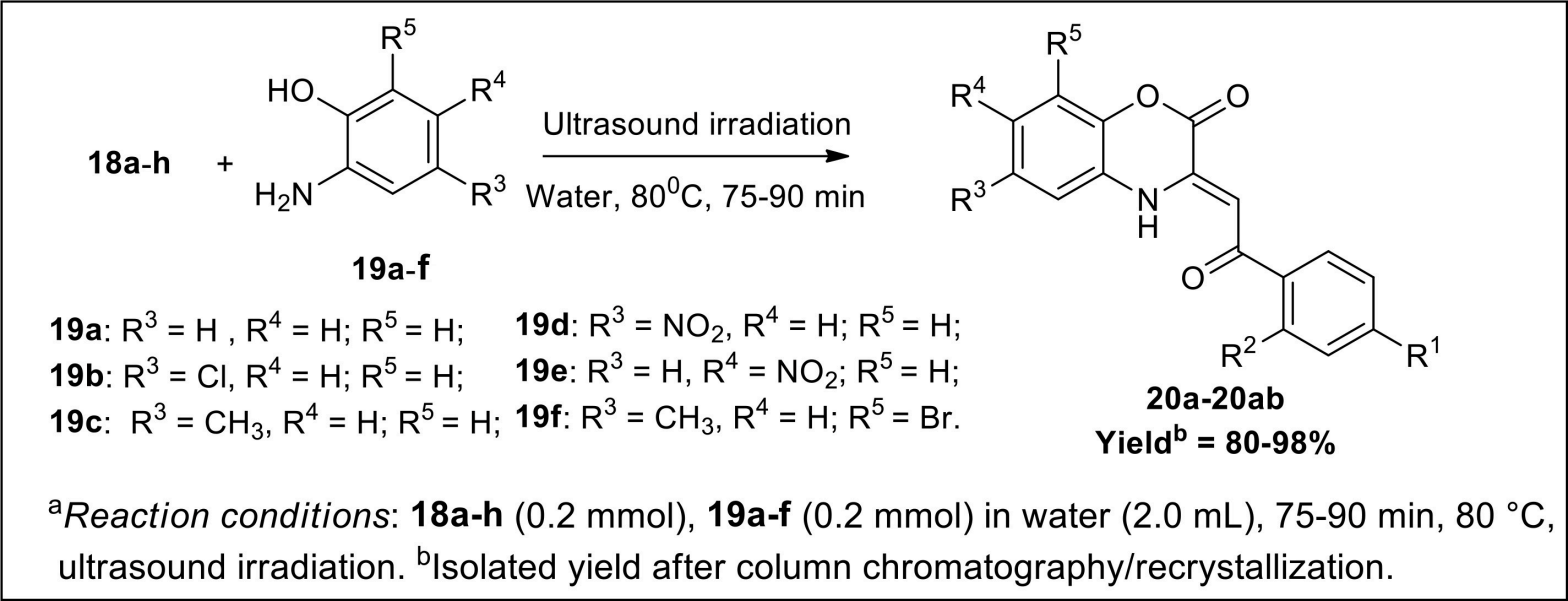
Ultrasound-assisted green synthesis of C-3 tethered 2-oxo-benzo [1,4]oxazine analogs **(20a-20ab)**.

The synthetic scheme for the synthesis of desired C-3 tethered 2-oxo-benzo[1,4]oxazine analogs **20a-20ab** using our reported procedure (Jaiswal et al., 2017) is depicted in Schemes 1, 2.

The base-mediated reaction of acetophenone **16a**-**h** with dimethyl oxalate in toluene for 6h furnished the diketo-ester **17a**-**h** in 70-80% yields. Conversion of these diketoesters **17a-h** to 2, 4-dioxo-4-phenylbutanoic acid **18a-h** were achieved by hydrolysis with LiOH.H_2_O in MeOH:THF:H_2_O (4:3:1) solvent. The reaction of nitro/alkyl/halide-substituted 2, 4-dioxo-4-phenylbutanoic acid **18a-h** with nitro/alkyl/halide-substituted 2-aminophenol **19a-f** in water furnished C-3 tethered 2-oxo-benzo[1,4]oxazines **20a**-**20ab** in 74-98% yields after purification either by flash column chromatography or by recrystallization method (Scheme 2, Figure 3; see Supplementary Figures 1–28 for details). All the synthesized compounds were well characterized by ^1^H-NMR and ^13^C-NMR spectroscopy, FTIR and HRMS analysis.

**Figure 3:**
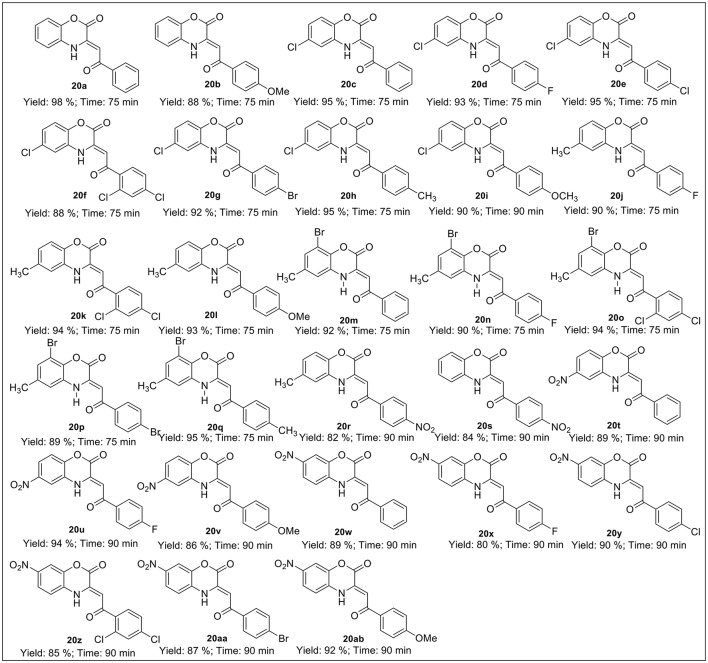
Structures of all synthesized C-3 tethered 2-oxo-benzo [1, 4]oxazines **(20a-20ab)**.

### Biological evaluation

#### DPPH radical scavenging antioxidant activity and SAR studies

All the synthesized C-3 tethered 2-oxo-benzo[1,4]oxazine analogs **20a-20ab** were evaluated for *in vitro* antioxidant activities using DPPH radical scavenging assay compared with standard reference ascorbic acid (Table 1). The choice of the reference compounds is based on hydrophilic nature of ascorbic acid and the maximum inhibition of the DPPH radical in IC_50_ value (μg/mL) by all the compounds **20a**-**20ab**. The DPPH radical scavenging assay is generally utilized as a quick and reliable parameter to investigate the antioxidant activities of diverse heterocycles (Baydar et al., 2007). DPPH is a stable free radical, that can easily accept a hydrogen radical or an electron to become a stable molecule (Blois, 1958). In the methanolic medium, DPPH has odd electron configuration having a strong absorption band at 515 nm, whereas this absorption decreases slightly in the presence of free radical scavengers, and it results color change to yellow from deep purple (Eklund et al., 2005; Sharma and Bhat, 2009). The radical trapping ability strongly depends on the structural availability of the radical trapping site. The steric hindrance as well as electron density plays a dynamic role in the antioxidant activity since they may prevent the test molecule from reaching the radical site of DPPH and thus results in low activity (Faria et al., 2006).

**Table 1:** Antioxidant activity of synthesized compounds **20a-ab** by DPPH radical scavenging assay and FRAP assay.

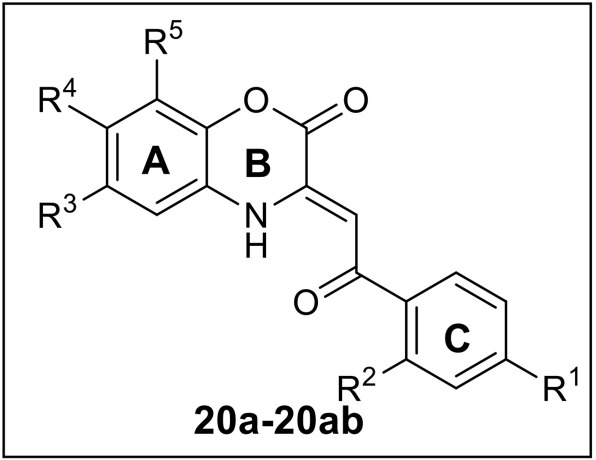								
**S. No**.	**Compound No**.	**R^1^**	**R^2^**	**R^3^**	**R^4^**	**R^5^**	**Antioxidant activity**^a^	
							**FRAP assay^b^ (C_0.5FRAP_ μM)**	**DPPH assay^c^ IC_50_ (μmg/mL)**
1	**20a**	H	H	H	H	H	611.5 ± 23.2	10.20 ± 0.08
2	**20b**	OCH_3_	H	H	H	H	686.4 ± 30.8	**6.89** ± **0.07**
3	**20c**	H	H	Cl	H	H	467.8 ± 22.4	28.80 ± 0.60
4	**20d**	F	H	Cl	H	H	732.7 ± 41.6	45.21 ± 0.92
5	**20e**	Cl	H	Cl	H	H	>1000	92.20 ± 1.54
6	**20f**	Cl	Cl	Cl	H	H	>1000	56.60 ± 1.12
7	**20g**	Br	H	Cl	H	H	798.6 ± 32.5	61.23 ± 1.23
8	**20h**	CH_3_	H	Cl	H	H	821.9 ± 38.7	44.83 ± 0.81
9	**20i**	OCH_3_	H	Cl	H	H	648.2 ± 29.5	34.94 ± 0.73
10	**20j**	F	H	CH_3_	H	H	502.6 ± 18.2	34.41 ± 0.70
11	**20k**	Cl	Cl	CH_3_	H	H	652.5 ± 30.6	43.58 ± 0.92
12	**20l**	OCH_3_	H	CH_3_	H	H	916.8 ± 21.4	43.80 ± 0.85
13	**20m**	H	H	CH_3_	H	Br	536.7 ± 21.4	24.38 ± 0.46
14	**20n**	F	H	CH_3_	H	Br	482.5 ± 35.5	16.86 ± 0.72
15	**20o**	Cl	Cl	CH_3_	H	Br	641.6 ± 28.7	22.48 ± 0.64
16	**20p**	Br	H	CH_3_	H	Br	>1000	44.32 ± 0.45
17	**20q**	CH_3_	H	CH_3_	H	Br	>1000	36.24 ± 0.27
18	**20r**	NO_2_	H	CH_3_	H	H	328.6 ± 25.8	12.23 ± 0.05
19	**20s**	NO_2_	H	H	H	H	618.4 ± 23.9	21.27 ± 0.28
20	**20t**	H	H	NO_2_	H	H	638.2 ± 32.6	**4.74** ± **0.08**
21	**20u**	F	H	NO_2_	H	H	424.5 ± 19.7	32.11 ± 0.52
22	**20v**	OCH_3_	H	NO_2_	H	H	>1000	43.58 ± 0.92
23	**20w**	H	H	H	NO_2_	H	597.4 ± 26.4	12.53 ± 0.09
24	**20x**	F	H	H	NO_2_	H	628.6 ± 32.4	10.18 ± 0.10
25	**20y**	Cl	H	H	NO_2_	H	708.2 ± 27.1	19.76 ± 0.35
26	**20z**	Cl	Cl	H	NO_2_	H	437.6 ± 39.4	28.81 ± 0.67
27	**20aa**	Br	H	H	NO_2_	H	518.6 ± 17.6	28.37 ± 0.16
28	**20ab**	OCH_3_	H	H	NO_2_	H	>1000	43.60 ± 0.74
29	**Ascorbic acid**	—	—	—	—	—	—	**4.57**
30	**BHT**	—	—	—	—	—	546.0 ± 13.6	—

a *Results are expressed as a mean ± standard deviation (n = 3). ^b^DPPH radical scavenging activities are expressed as IC_50_ concentrations of the compounds (μg/mL) required to inhibit 50% of the radicals and the maximum inhibition values and Positive control for DPPH assay=Ascorbic acid; ^c^Positive control for FRAP assay=BHT. The bold value indicates promising antioxidant compounds*.

Kareem et al. proposed two mechanisms involved in DPPH assay; first one is the hydrogen atom transfer (HAT) mechanism and the second one is the single electron transfer (SET) mechanism (Kareem et al., 2015). Similar to their interpretation, it can be speculated that, for DPPH assay, a dominant HAT mechanism is assumed and the favored hydrogen abstraction sites are enamine –NH group, preferably with conjugation to the side chain of phenacyl group (-COPh), as the latter could stabilize by the resulting radical from additional resonance structures.

**Figure 4:**
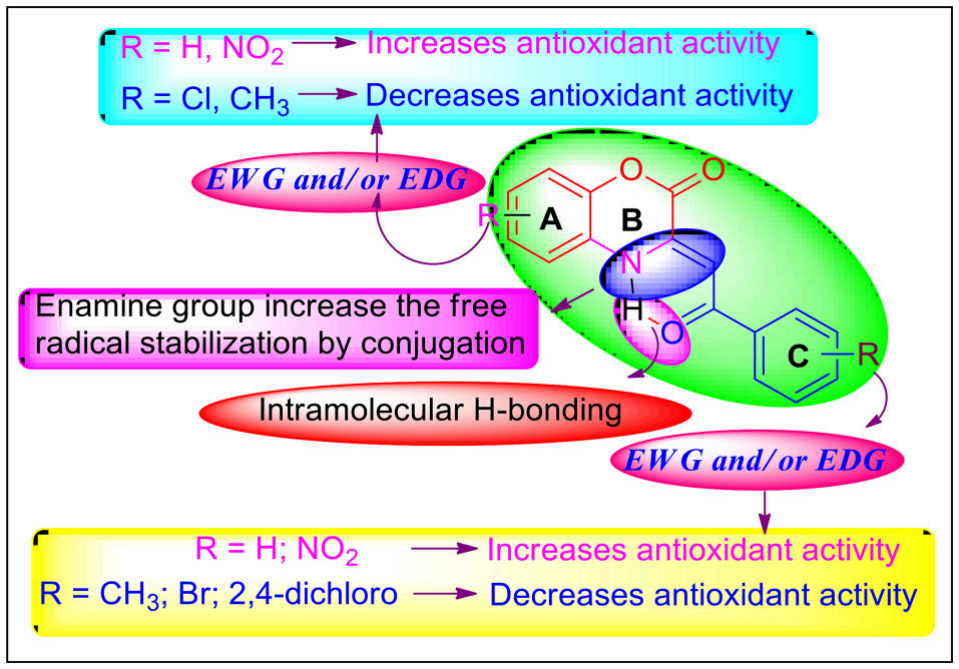
SAR analysis of synthesized 2-oxo-benzo[1,4]oxazines.

The generic scaffold of the newly synthesized C-3 tethered 2-oxo-benzo[1,4]oxazines, as illustrated in Figure 4, consists of a two fused cyclic ring A and B linked with ring C *via* α, β-unsaturated ketone having electron-withdrawing group (EWG) and/or electron-donating group (EDG) either at ring A or at C. The active group -CO-C=C-NH- enables resonance between the ring B and C, leading to multiple resonance structure, which may be further initiated by the attached substituents of ring A and C and in situ enhances the radical scavenging activity through the removal of hydrogen atom from NH of ring B *via* HAT mechanism. It has been found that electron withdrawing substituent NO_2_ at ring A or C increase the antioxidant activity which may be due to resonance based stabilizing effects. Therefore, based on the structures and their antioxidant activities, it was found that the compounds have either no substitution at ring A and C or have EWG/EDG at ring A and C plays a very important role in deciding their DPPH radical scavenging activities. Hence, based on the substituent's (either EWG or EDG) at ring A and C of 2-oxo-benzo[1,4]oxazines **20a-20ab**, and their antioxidant activities, their structure-activity relationship can be explained by grouping all compounds into two groups:

- **No substitution or EDG at ring A or ring C**: In the first group of compounds having no substitution at ring **A** and **C** i.e., the model compound **20a**, exhibited promising antioxidant activity (IC_50_ = 10.20 ± 0.08 μg/mL) in comparison with standard reference Ascorbic acid (IC_50_ = **4.57** μg/mL) [entry 1]. Then, by putting EDG (OMe group) at ring C, as in compound **20b**, further increases activity (IC_50_ = 6.89 ± 0.07 μg/mL) [entry 2]. Reversing the order i.e., halogen substitution at ring **A** and no substitution at ring C do not cause any further increase in antioxidant activity as shown by compound **20c** (entry 3). Furthermore, when we incorporated halogen substituents (Cl, F, Br, 2,4-dichloro) or CH_3_ substituent either at A or C as in the case of compounds **20c-l**; a decrease in the antioxidant activity was observed due to high electron density in compounds **20d**-**g, 20j** and **20k** (entry 4-12). Furthermore, It was observed that two EDG at ring A (**20m-q**; entry 13-17) exhibited moderate antioxidant activity having IC_50_ value in the range of 18.86 ± 0.72 to 44.32 ± 0.45 μg/mL. In this series (**20m-q**; entry **13-17**), ring C having Fluorine substituent i.e. compound **20n** exhibited good antioxidant activity (IC_50_ = 16.86 ± 0.72 μg/mL) in comparison with other compounds (**20m** and **20o**-**q**).
  To our surprise; when we incorporated EWG group i.e., NO_2_ group at ring C and EDG group i.e., CH_3_ group at ring A (compound **20r**); the antioxidant activity was regained and shows IC_50_ value of 12.23 ± 0.05 μg/mL nearly equivalent to **20a** (entry 18).
  It is to be noted that EDG at ring A decreases antioxidant activity as shown in entries 3-17; so, we synthesized compound **20s** (having no any substitution at ring A and EWG i.e., NO_2_ group at ring C), which, contrary to our expectations, displayed further decrease in antioxidant activity (IC_50_ = 21.27 ± 0.28 μg/mL) (entry 19).
- **EWG (-NO**_2_ **Group) at ring A**: Since **20r** having EWG (NO_2_) at ring C showed promising antioxidant activity; inspired by this observation, we prepared **20t**-**v** having NO_2_ group at C-4 position of ring A. 2-oxo-benzo[1,4]oxazine **20t** having no substitution at ring C, showed excellent antioxidant activity having IC_50_ value of 4.74 ± 0.08 μg/mL (entry 20). Since **20b** having OMe substituent at ring C, was also found to show excellent antioxidant activity; thus, we synthesized **20u** and **20v** having Fluoro as well as OMe substituent, respectively at ring C. Unfortunately, antioxidant activity diminishes (entry 21 and 22). In addition, we also prepared 2-oxo-benzo[1,4]oxazines **20w**-**20ab** having EWG group (NO_2_) at C-5 position of ring A further to investigate SAR study. Compound **20w** having no substitution at ring C showed promising antioxidant activity having IC_50_ value of 12.53 ± 0.09 μg/mL (entry 23). On incorporating Fluoro group at ring C; activity of **20x** increases (IC_50_ = 10.18 ± 0.10 μg/mL, entry 24). Furthermore, when we incorporated halogen substituents (Cl, Br, 2,4-dichloro and OMe) at ring C as in the case of compounds **20y**-**20ab**; a decrease in the antioxidant activity was observed (entry 19-23).
  Overall, we can interpret that no substitution or EWG at ring A or ring C enhances antioxidant activity of all the synthesized 2-oxo-benzo[1,4]oxazines **20a**-**20ab**. Whereas EDG either at ring A or C diminishes antioxidant activity. Our SAR results depict that **20b** and **20t**, the best compounds of the series, showed antioxidant activity comparable to standard reference Ascorbic acid.

### Ferric reducing antioxidant power (FRAP) activity and SAR studies

The FRAP assay was deliberated using the method as illustrated by Benzie and Strain (Benzie and Strain, 1996). It reveals that the reducing potential of an antioxidant molecule, which reacts with a complex of ferric tripyridyltriazine [Fe^3+−^TPTZ] and develops a colored ferrous tripyridyltriazine [Fe^2+^-TPTZ]. The reducing nature of an antioxidant depends on their property to donate a hydrogen atom for the breaking of the free radical chain, which is responsible for oxidative stress etc.

All the synthesized C-3 tethered 2-oxo-benzo[1,4]oxazine analogs **20a-20ab** were assessed for FRAP assay taking BHT as standard reference; as depicted in Table 1. In this study, the trend with respect to ferric ion reducing activities of all the screened compounds i.e., **20a-20ab** showed that eight compounds (**20c**, **20j**, **20m**, **20n**, **20r**, **20u**, **20z**, and **20aa**) were found more potent than BHT (**C**_0.5FRAP_ = 546.0 ± 13.6 μM).

In summary, all the compounds (**20a**-**20ab**) displayed good to moderate activity in comparison with BHT in the range of C_0.5FRAP_ = 328.6 ± 25.8 μM to 916.8 ± 21.4 μM in *in-vitro* antioxidant FRAP assay except compounds **20e**, **20f**, **20l**, **20q, 20v**, and **20ab** which showed C_0.5FRAP_ greater than 1000 μM. Compounds having EWG i.e. NO_2_ substituent at ring C (**20r** and **20s**) showed different potency than standard reference BHT. While **20r** having CH_3_ at C-4 position of ring A displayed potent activity than BHT; **20s** was found to be less active. Anomaly was observed in the case of compounds having no substitution at ring C. While **20c** and **20m** showed greater potency; compounds **20a, 20t** and **20w** were found less active than BHT.

### Cell toxicity study

**Figure 5:**
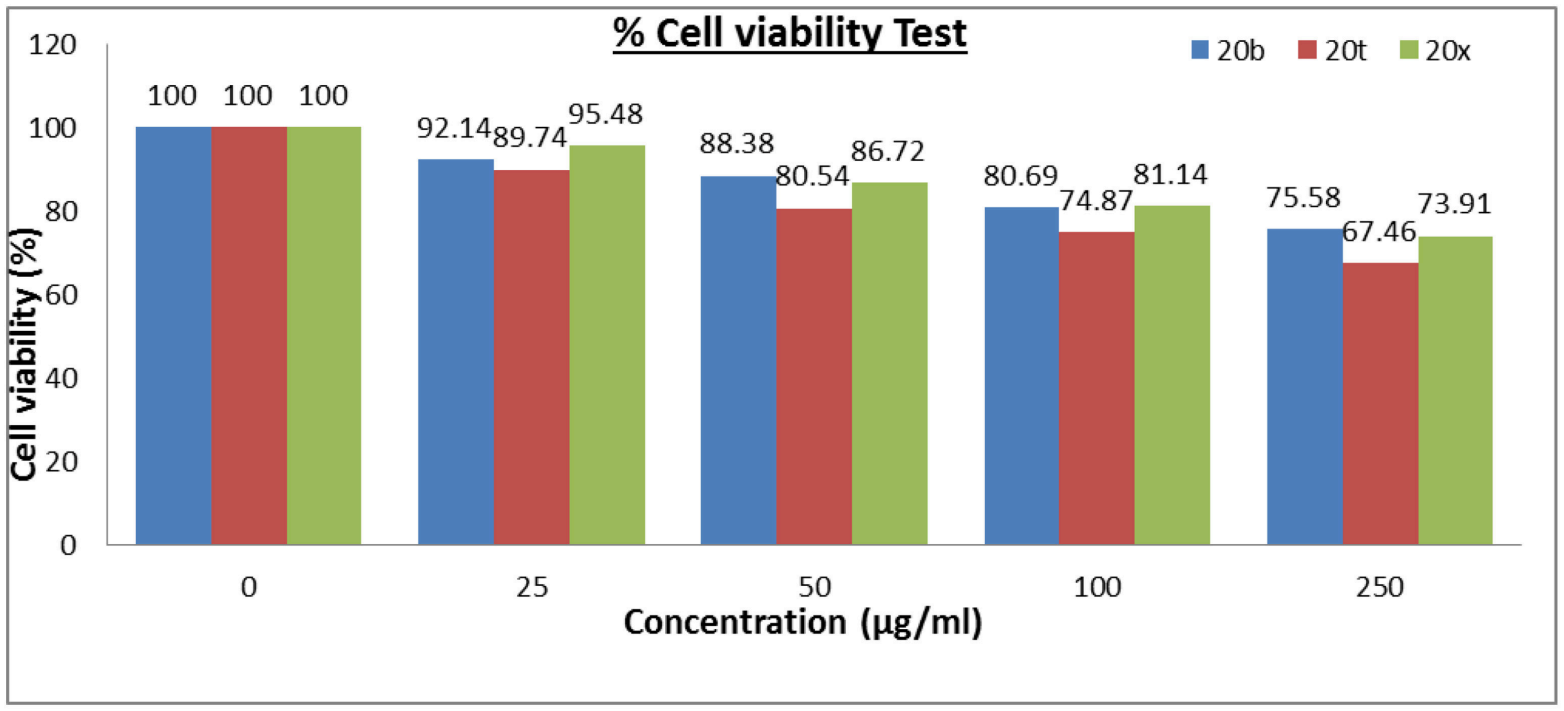
Percentage cell viability test.

Out of 28 compounds, three most active compounds i.e., **20b**, **20t**, and **20x** were then selected for their cytotoxic studies. As depicted in Figure 5, compounds **20b**, **20t**, and **20x** were accessed for their cytotoxic study using 3T_3_ fibroblast cell lines in MTT assay (Danihelová et al., 2013). The result showed that these compounds were non-toxic in nature (>65% cell viability) even at 250 μg/mL concentration and therefore, displays permissible values of cell viability.

### *In silico* molecular docking simulation studies

Finally, the biological results were validated *via in silico* molecular docking studies of two most active compounds (**20b** and **20t**). Since **20b** has OMe group at ring C and **20t** has NO_2_ group at ring A; it is worthwhile to compare the docking studies of active compounds with molecules having no substitution either at ring A or C. Therefore, we have also selected compound **20a** for our *in silico* molecular docking simulation studies. For that purpose, Peroxiredoxins (Prdxs), a family of small human antioxidant enzyme, was selected as our target protein. Peroxiredoxins contain essential cysteine residues as catalyst and thioredoxin as an electron donor, which help in scavenging peroxide and are involved in the metabolic cellular response to ROS (Neumann et al., 2003; Monteiro et al., 2007).

The binding affinities and interactions of C-3 tethered 2-oxo-benzo [1, 4] oxazine derivatives with the human antioxidant enzyme were investigated through molecular docking simulations. Binding affinities were predicted by the Sybyl docking total score upon docking with the Surflex-Dock program (Sybyl X 2.0).Compounds were docked into the active site of the known the human antioxidant enzyme target peroxiredoxins (Prxs) DTT complex (PDB ID: 3MNG) were taken from the Protein Data Bank (http://www.rcsb.org/pdb) (Hall et al., 2010b; Bayoumi et al., 2012; Yadav et al., 2014a,b; Yapati et al., 2016).

Docking studies were carried out to evaluate the binding affinity and interactions with their target proteins. Hydrogen bonds (H-bonds, with a donor-receptor distance of 3Å) between the ligand and amino acids in the binding site of the protein were used for the ranking of compounds. The mode of interaction of the co-crystallized ligand dithiothreitol (DTT) within the crystal structure of enzyme in complex was used as a reference binding model. The root mean-square deviation (RMSD) of each docking pose was compared to the co-crystallized ligand and used for ranking and for RMSD calculation. The co-crystallized DTT molecule was re-docked onto the same binding site and the most probable binding mode was selected as that with the highest docking total score of 4.8921. An RMSD value 0.6772Å between the predicted and crystal binding mode indicates the high reliability of Surflex-Dock for this protein target.

**Figure 6:**
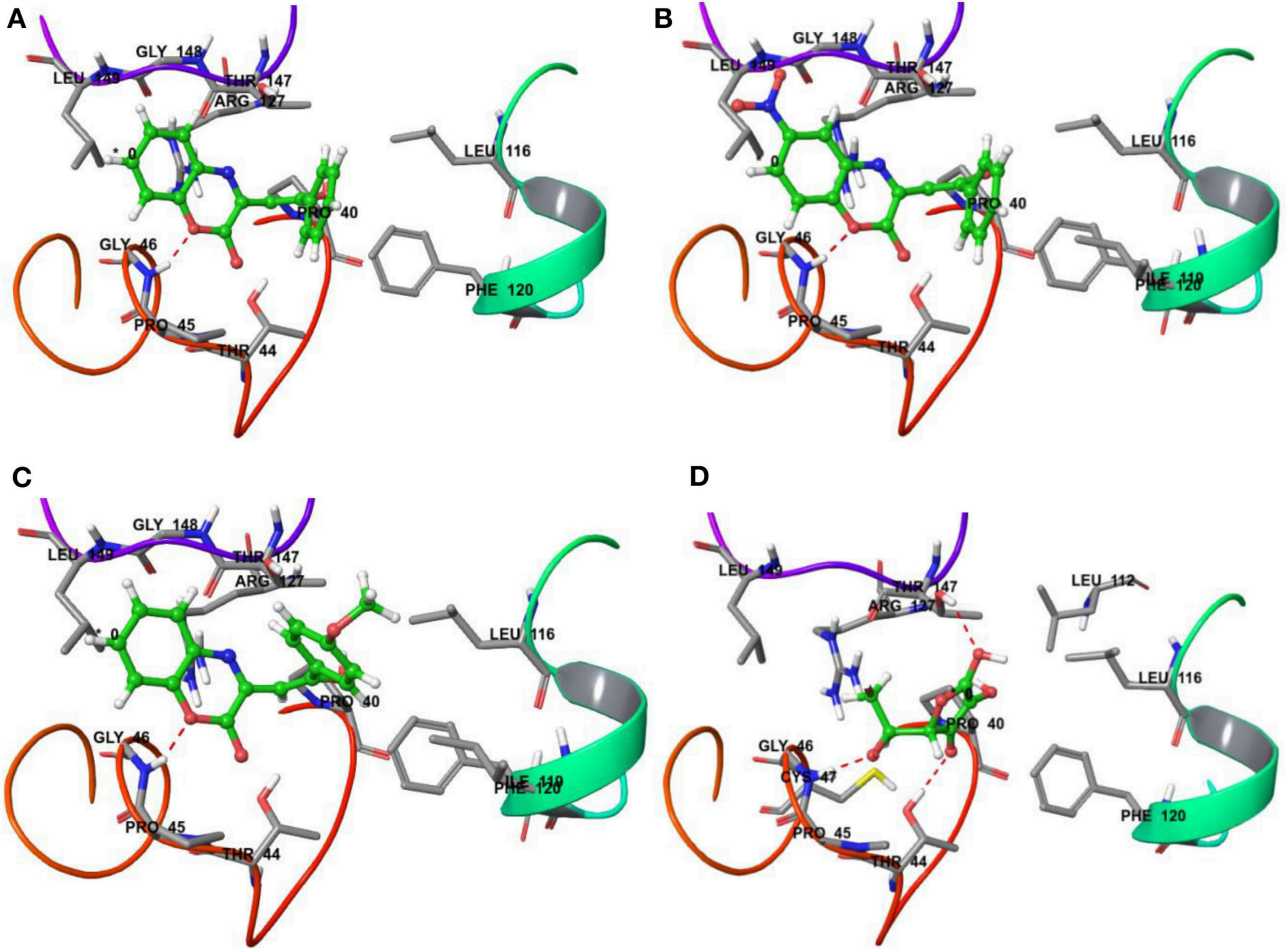
Binding interactions of compound **20a, 20b, 20t** and reference drug Ascorbic acid upon docking onto human antioxidant enzyme target peroxiredoxins (Prxs) (PDB ID: 3MNG). The formation of a H-bond of length 1.8Å to residue Gly-46 in the binding site was predicted in the case of **20a, 20b, and 20t** along with the formation of four H-bond of length 2.2, 1.8 and 2.1 Å to residue Thr-147, Gly-46 and Thr-44 in the binding site was predicted in the case of reference drug Ascorbic acid. **(A)** A top docking energy (total score) of 3.8470 was predicted for **20a**; **(B)** A top docking energy (total score) of 3.6567 was predicted for **20b**. **(C)** A top docking energy (total score) of 4.2709 was predicted for **20t**. **(D)** A top docking energy (total score) of 3.4829 was predicted for Ascorbic acid.

On the other hand, docking results for **20a, 20b**, and **20t** against the antioxidant target protein Prxs showed a high binding affinity docking score indicated by a total score of 3.8470 (Figure 6A), 3.6567 (Figure 6B) and 4.2709 (Figure 6C) forms a H-bond (NH_2_…O) of length 1.8Å to the backbone of hydrophobic aliphatic residue that is, Glycine-46. In the docking pose of the **20a, 20b**, and **120t** and Prxs complex, the chemical nature of binding site residues within a radius of 3Å with diverse properties was aromatic (hydrophobic), for example, Phe-120, (Phenylalanine); hydrophobic, for example, Leu-116, Ile-119, Leu-149, Leu-112(Leucine), Gly-46, Gly-148(Glycine); (polar, hydrophobic, positive charged) residues, for example, Arg-127 (Arginine); nucleophilic (polar, hydrophobic), for example, Thr-147 and Thr-44 (Threonine), nucleophilic (polar uncharged), for example Cys-47 (Cysteine); and hydrophobic (polar, uncharged) residues, for example, Pro-40 and Pro-45 (Proline) as a result, the bound compound showed a strong hydrophobic interaction with Prxs, thus leading to more stability and activity in this compound.

The docking results for the ascorbic acid (standard compound) with the antioxidant target protein Prxs showed a low binding affinity docking score, indicated by a low total score of 3.4829 with three H-bond (hydrogen bond) formation of length 2.2, 1.8 and 2.1Å to the Thr-147, Gly-46 and Thr44 (Figure 6D). The ascorbic acid-Prxc-docked complex also showed a similar type of binding site residues within a radius of 3Å of bound ligand such as Thr-147, Leu-116, Pro-40, Phe-120, Leu-112, Thr-44, Gly-46, Pro-45, Cys-47, Arg-127, Leu-149 shown in Figure 6D. Thus, the docking procedure of Surflex-dock software (Sybyl-X 1.3) in reproducing the experimental binding affinity seems reliable, and therefore predicted as true positive.

Thus, it can be inferred based on docking simulation studies that the most active compounds i.e., **20b** and **20t** having IC_50_ value of 6.89 ± 0.07 μg/mL and 4.74 ± 0.08 μg/mL, showed the binding affinity docking score of 3.6567 and 4.2709, respectively (Figures 6B,C), which were found to be comparable to the binding affinity docking score of standard reference ascorbic acid (Figure 6D). While comparing the binding energy docking score of **20a** (unsubstituted both at ring A and C) i.e., 3.8470 (Figure 6A) with ascorbic acid and the two most active compounds; the results were also found to be comparable. Thus, the *in silico* docking results of **20b** and **20t** successfully validated the *in vitro* experimental studies.

## Conclusion

In summary, we disclose C-3 tethered 2-oxo-benzo [1, 4]oxazine analogs **20a-20ab** as a of potent antioxidant agents. Compound **20b** and **20t**, the most active compounds of the series, showed promising antioxidant activity having IC_50_ value of 6.89 ± 0.07 μg/mL and 4.74 ± 0.08 μg/mL, respectively, in DPPH radical scavenging assay in comparison with ascorbic acid (IC_50_ = 4.57 μg/mL). Whereas in FRAP assay, eight compounds (**20c**, **20j**, **20m**, **20n**, **20r**, **20u**, **20z**, and **20aa**) were found more potent than BHT (**C**_0.5FRAP_ = 546.0 ± 13.6 μM). The active compounds were also found non-toxic in 3T_3_ fibroblast cell lines in MTT assay. Our *in silico* molecular docking results reveal that **20b** and **20t** showed excellent docking total scores against human antioxidant enzyme target as compared to ascorbic acid. Thus, the *in silico* docking simulation studies effectively validated the *in vitro* experimental results.

## Author contributions

SC was responsible for the study of concept and design of the project. VS and PKJ were responsible for the performing synthetic reactions, acquisition and analysis of data. MS, MM, and AKS were responsible for the pharmacological *in vitro* activity evaluation of synthesized compounds. DKY and Saloni performed docking studies. SM and MHK provided molecular modeling facility. SC and PKJ drafted the manuscript. All authors read and approved the final manuscript.

### Conflict of interest statement

The authors declare that the research was conducted in the absence of any commercial or financial relationships that could be construed as a potential conflict of interest.
