# Supplementary material for: Discovery of C-3 Tethered 2-oxo-benzo[1,4]oxazines as Potent Antioxidants: Bio-Inspired Based Design, Synthesis, Biological Evaluation, Cytotoxic, and in Silico Molecular Docking Studies
Source: Front Chem. 2018 Mar 23;6:56. doi: 10.3389/fchem.2018.00056 (PMC5876303; doi:10.3389/fchem.2018.00056)
Supplement: Supplementary file 1 [file Image1.pdf]

Supplementary Material

Discovery of C-3 tethered 2-oxo-benzo[1,4]oxazines as potent antioxidants: Bio-inspired based design, synthesis, biological evaluation, cytotoxic and Insilico molecular docking studies

Vashundhra Sharma,[1,‡] Pradeep K. Jaiswal, [1,‡] Mukesh Saran,[2] Dharmendra K. Yadav,[3,*] Saloni,[3] Manas Mathur,[2] Ajit K. Swami,[2] Sanjeev Misra,[4] Mi-hyun Kim,[3] and Sandeep Chaudhary,[1,*]

[1]Laboratory of Organic and Medicinal Chemistry, Department of Chemistry, Malaviya National Institute of Technology, Jawaharlal Nehru Marg, Jaipur-302017, India.

[2]Department of Advance Molecular Microbiology, Seminal Applied Sciences Pvt. Ltd. Jaipur-302015, India.

[3]College of Pharmacy, Gachon University of Medicine and Science, Hambakmoeiro 191, Yeonsu-gu, Incheon city, 406-799, Korea.

[4]Department of Biochemistry, All India Institute of Medical Sciences (AIIMS), Jodhpur, Rajasthan-342005, India.

[‡]*Both authors have equal contribution.*
*** Correspondence:**
Corresponding Author: Sandeep Chaudhary
E-mail: schaudhary.chy@mnit.ac.in
Dharmendra K. Yadav
Email: dharmendra30oct@gmail.com

Contents	Page No.
Copies of characterization spectra of ^{1}H and ^{13}C NMR of C-3 tethered 2-oxo-benzo[1,4]oxazines **(20b, 20j-t, 20x and 20z)**	**S2-S15**
MTT Assay	**S16**

Keywords: 2-Oxo-benzo[1,4]oxazines; Antioxidant; DPPH, FRAP, Ascorbic acid, BHT
[*]Corressponding author. Tel: +82-32-820-4947; E-mail: dharmendra30oct@gmail.com
[*]Corressponding author. Fax.: 911412529029; Tel: +91-141-2713319; E-mail: schaudhary.chy@mnit.ac.in

Representative characterization spectra (^{1}H and ^{13}C NMR) of C-3 tethered 2-oxo-benzo[1,4]oxazines (20b, 20j-t, 20x and 20z):

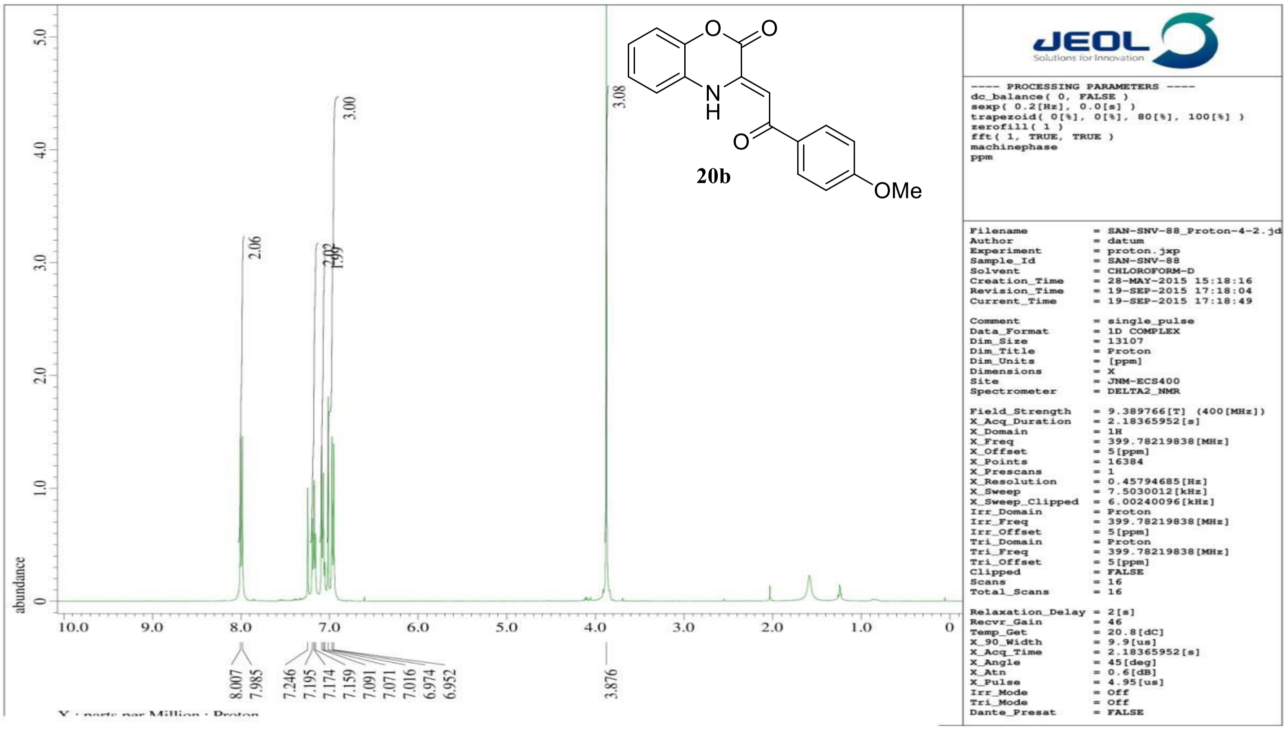

Figure 1. ^{1}H NMR Spectra of Compound **20b**.

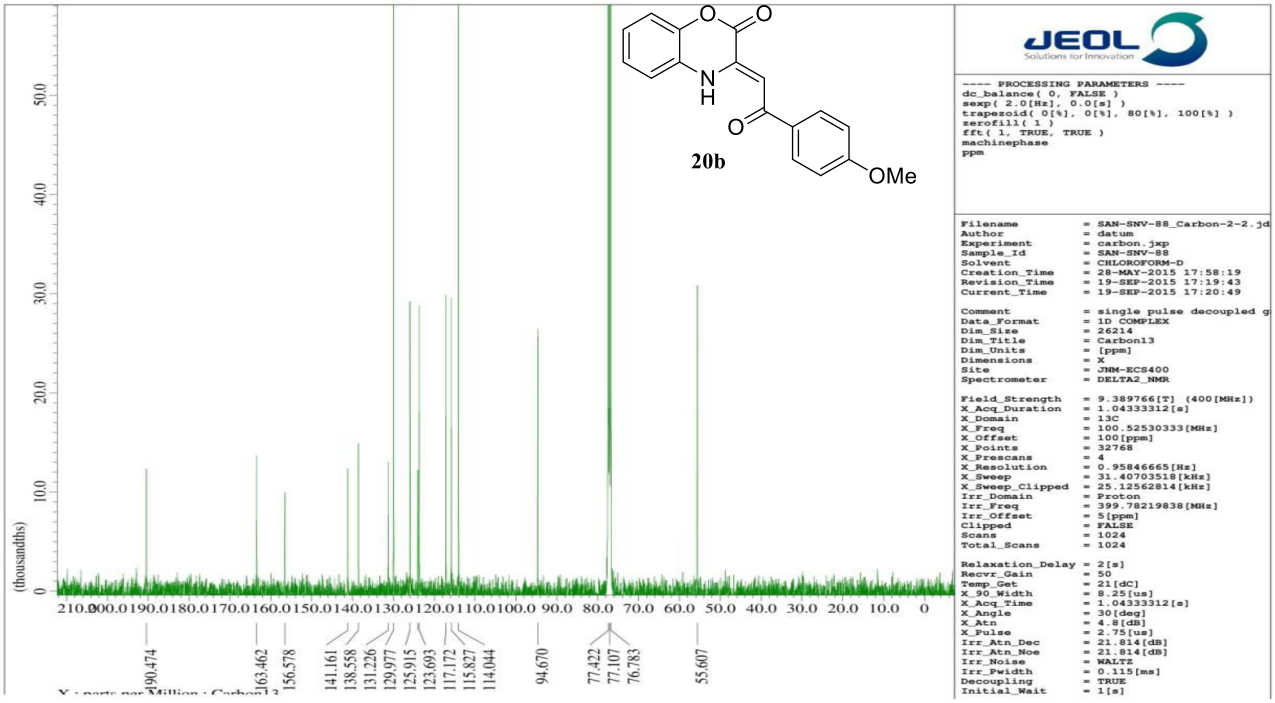

Figure 2. ^{13}C NMR Spectra of Compound **20b**.

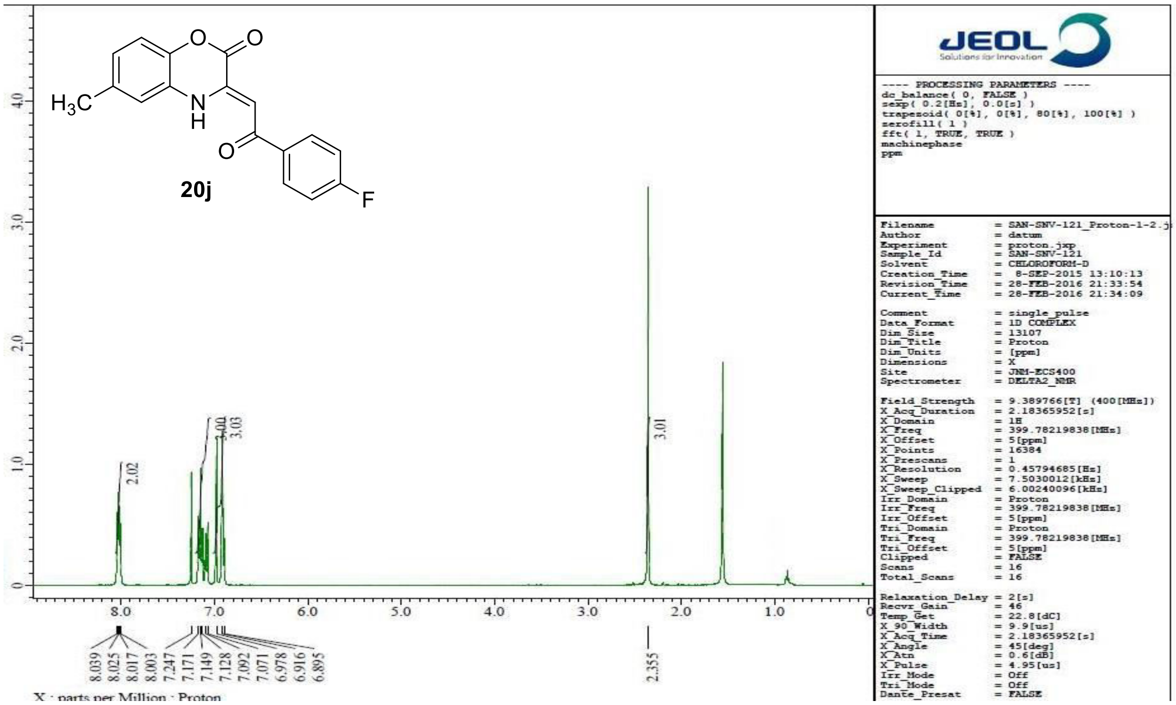

Figure 3. ^{1}H NMR Spectra of Compound **20j**.

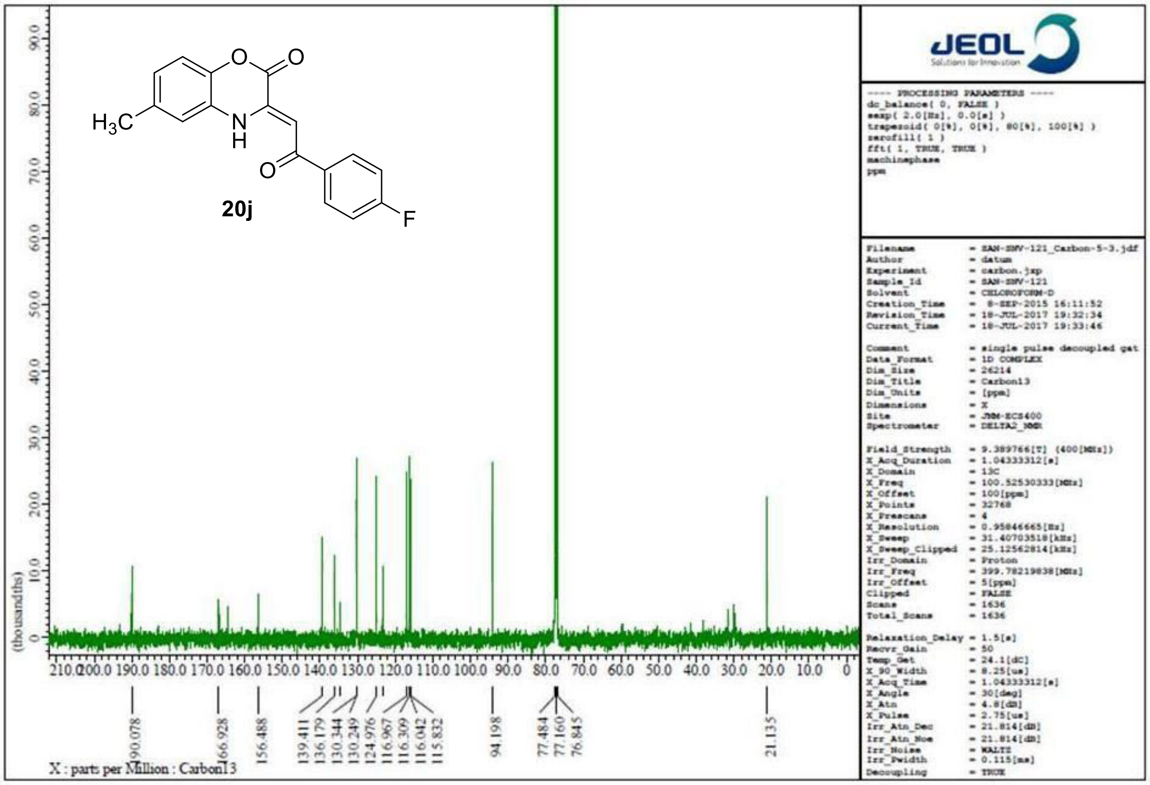

Figure 4. ^{13}C NMR Spectra of Compound **20j**.

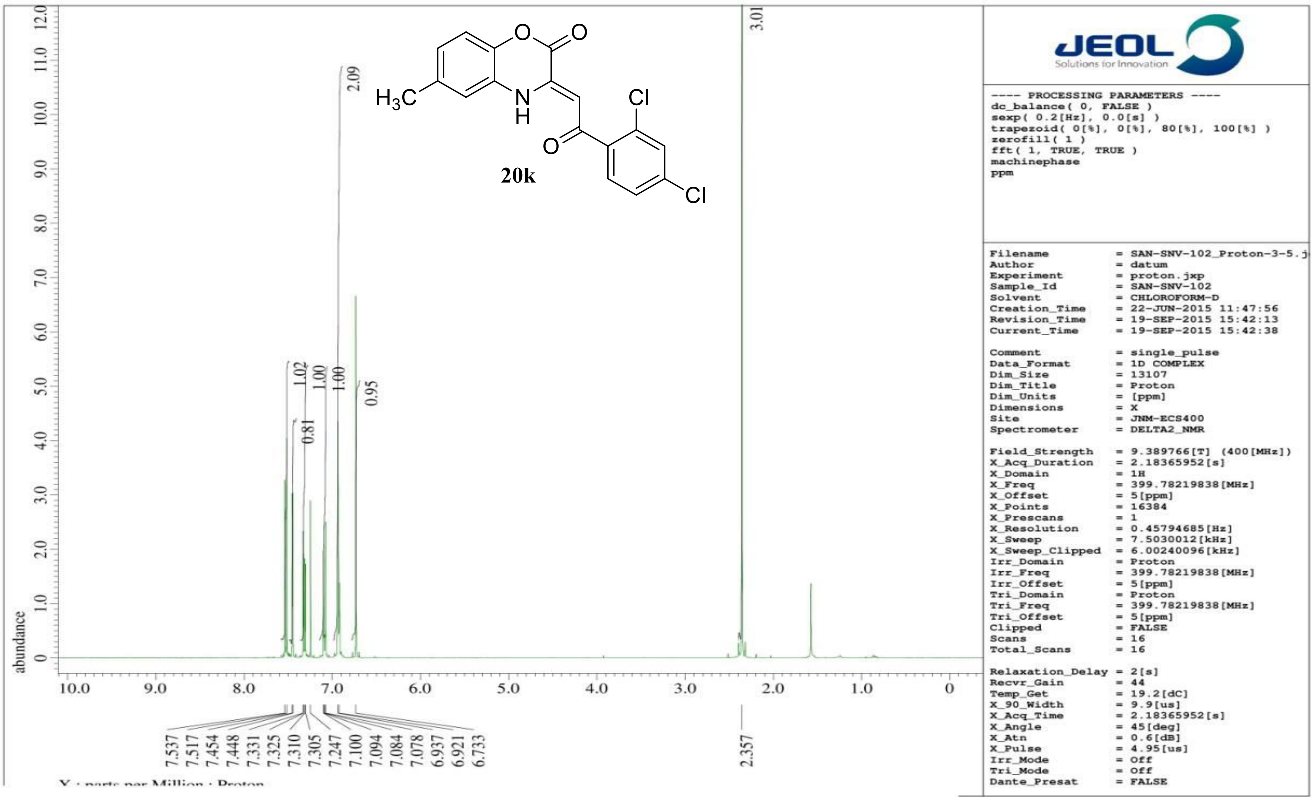

Figure 5. ^{1}H NMR Spectra of Compound **20k**.

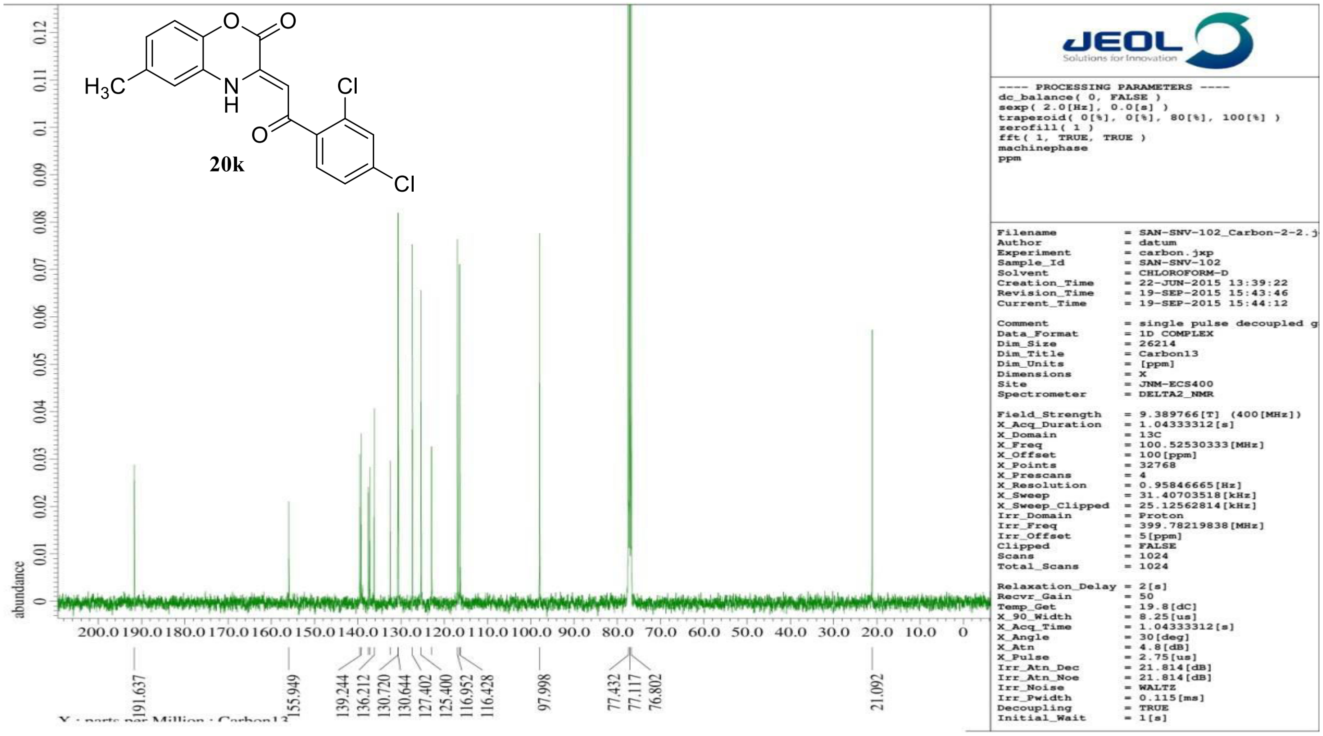

Figure 6. ^{13}C NMR Spectra of Compound **20k**.

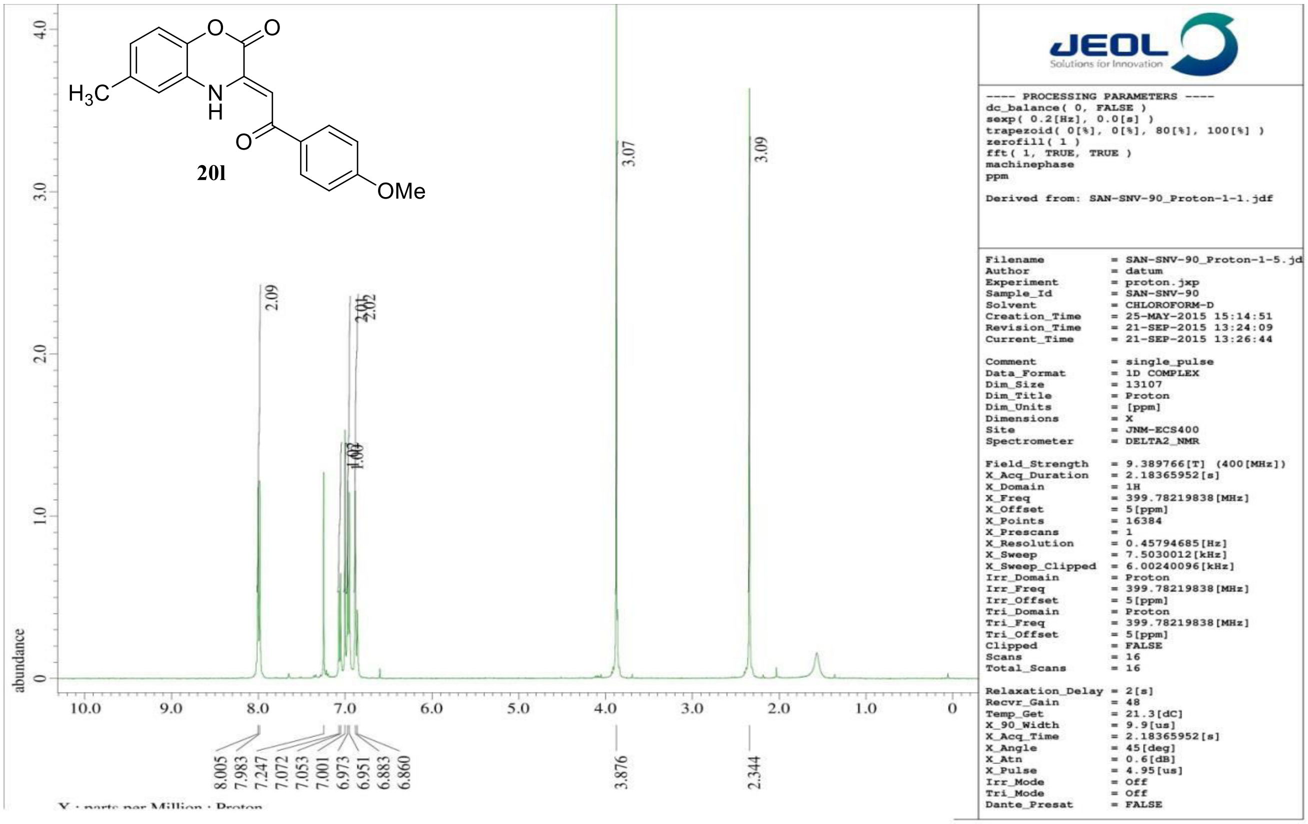

Figure 7. ^{1}H NMR Spectra of Compound **20l**.

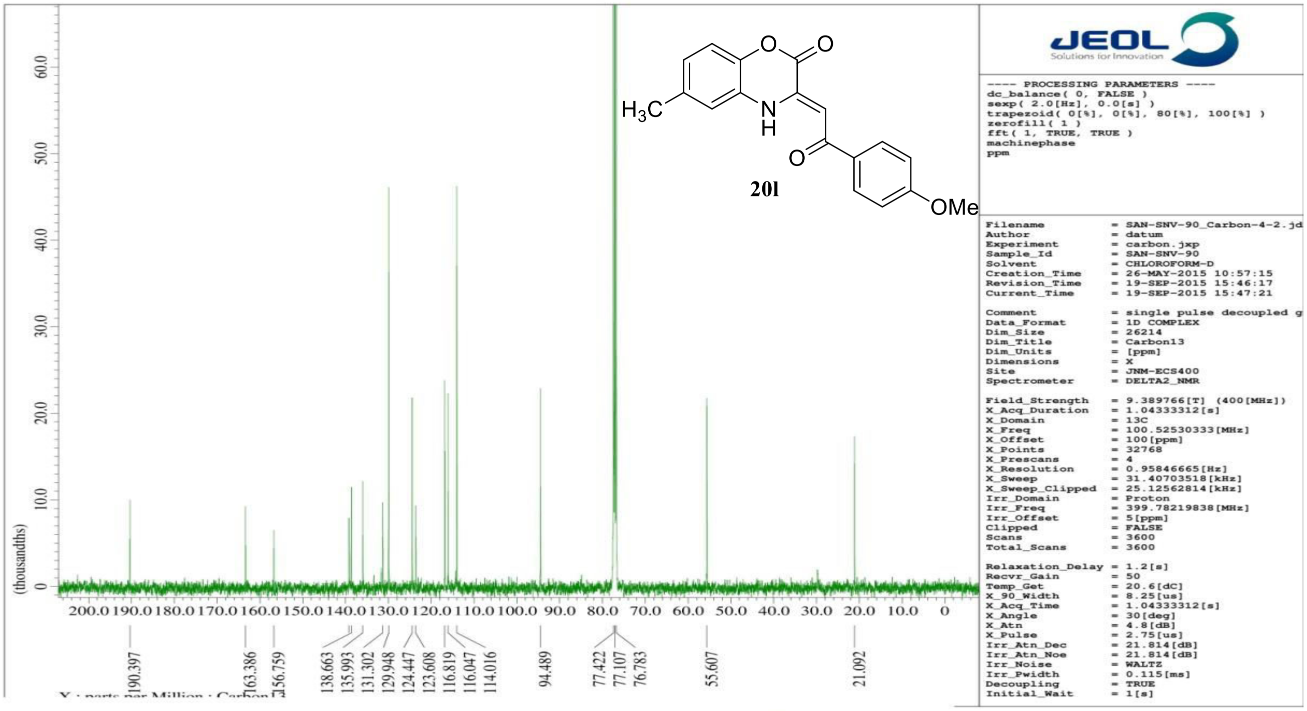

Figure 8. ^{13}C NMR Spectra of Compound **2ol**.

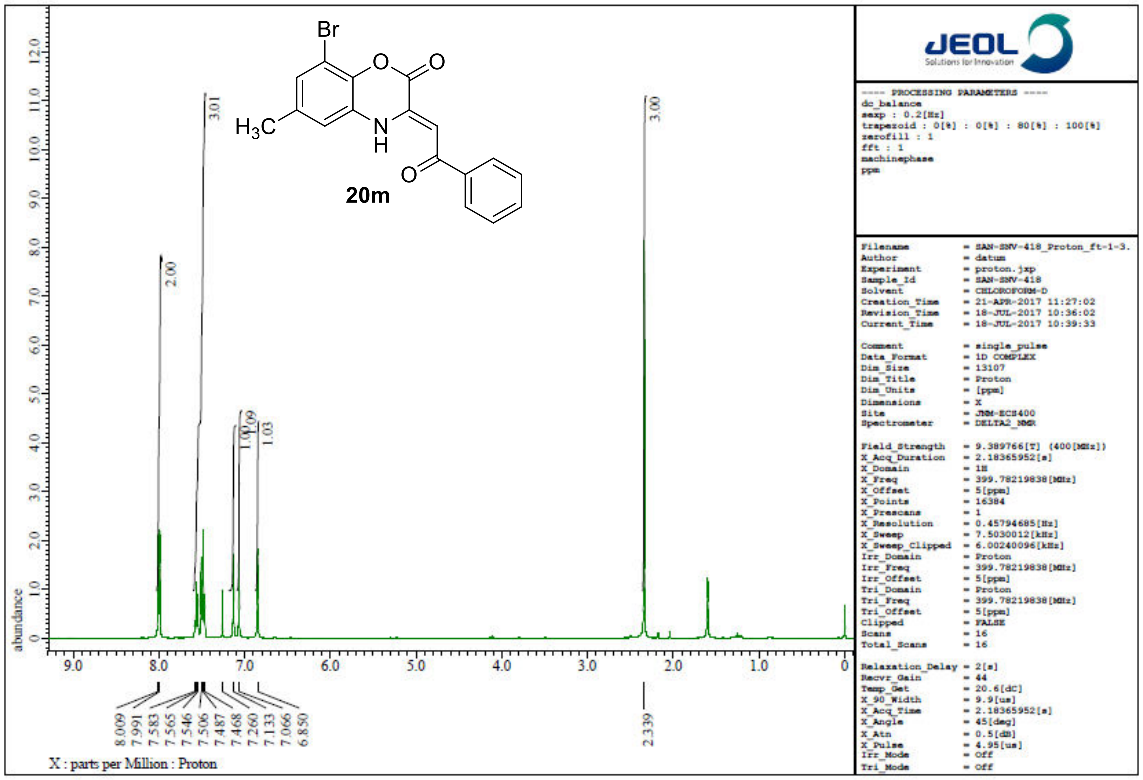

Figure 9. ^{1}H NMR Spectra of Compound **20m**.

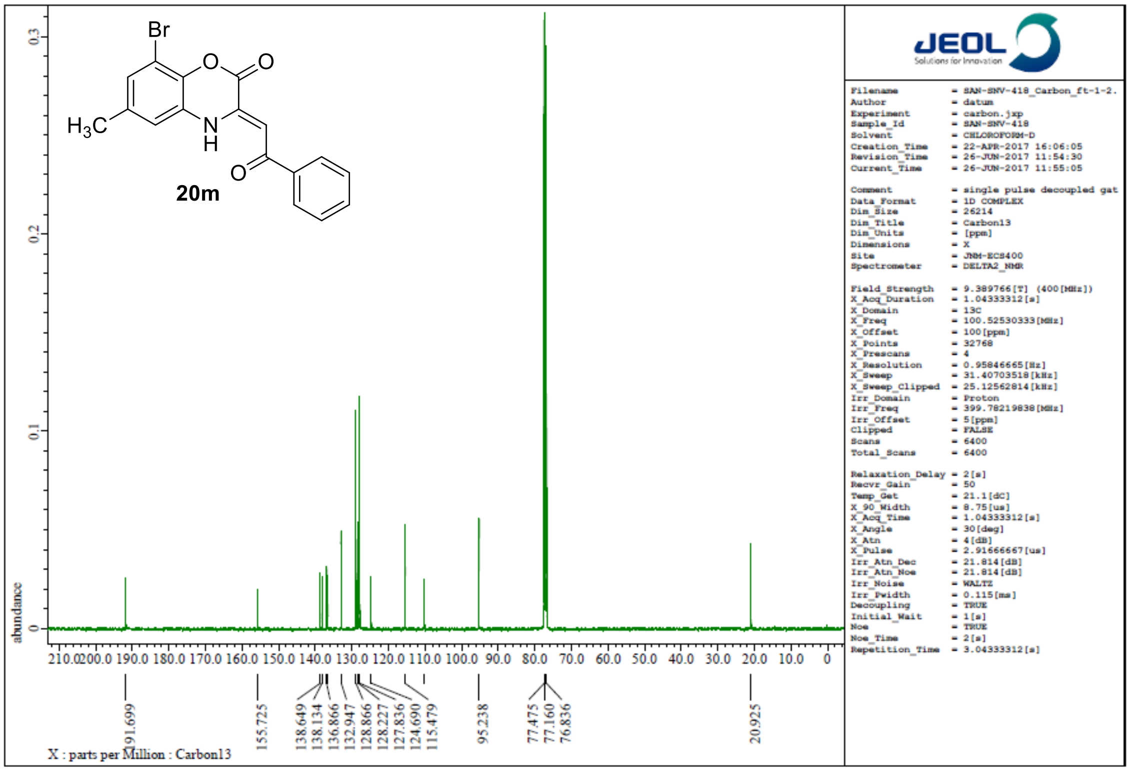

Figure 10. ^{13}C NMR Spectra of Compound **20m**.

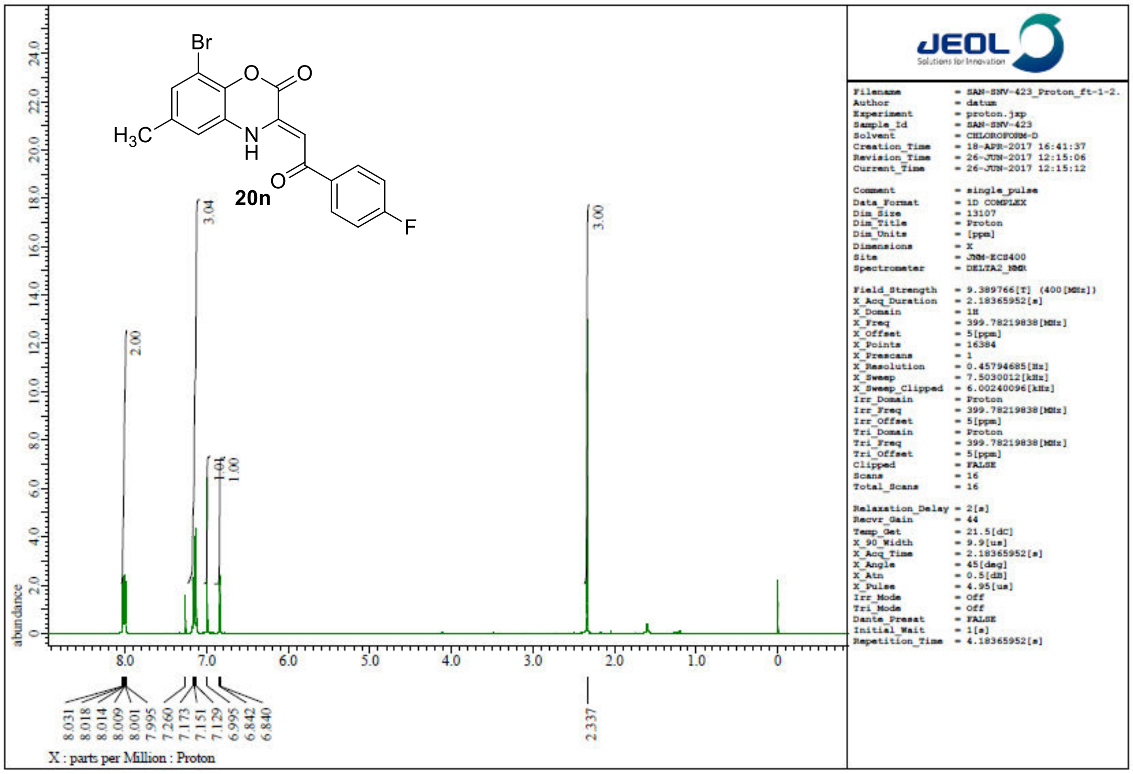

Figure 11. ^{1}H NMR Spectra of Compound **20n**.

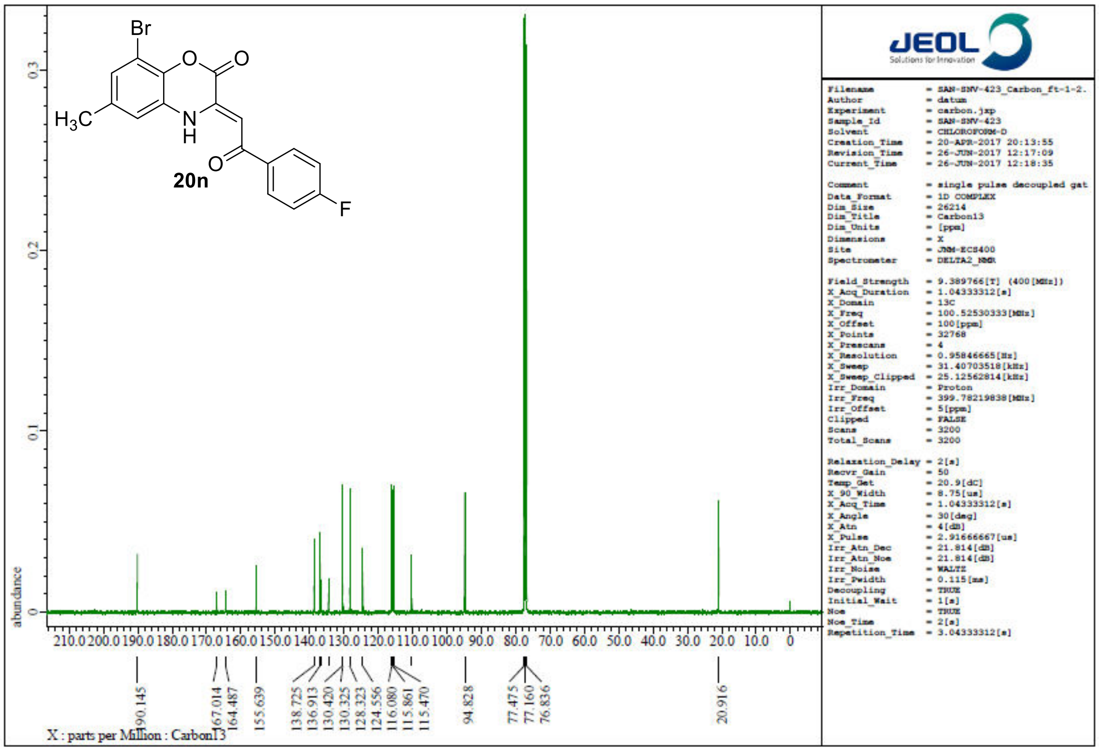

Figure 12. ^{13}C NMR Spectra of Compound **20n**.

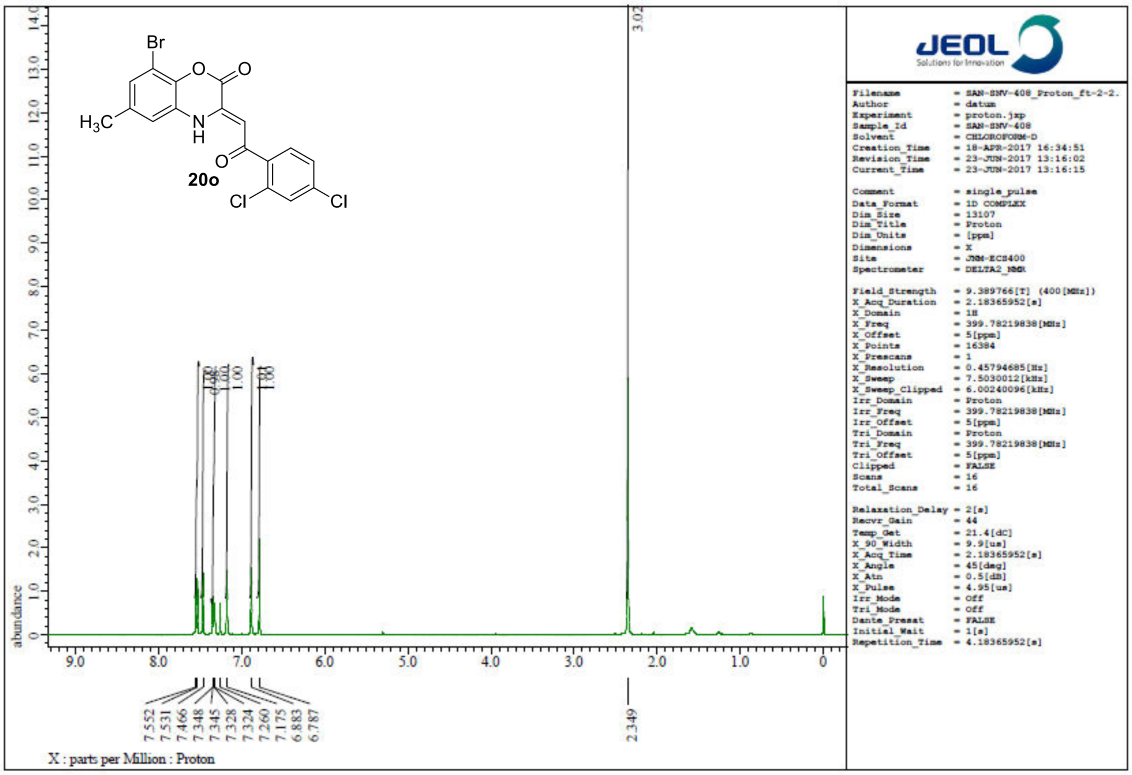

Figure 13. ^{1}H NMR Spectra of Compound **20o**.

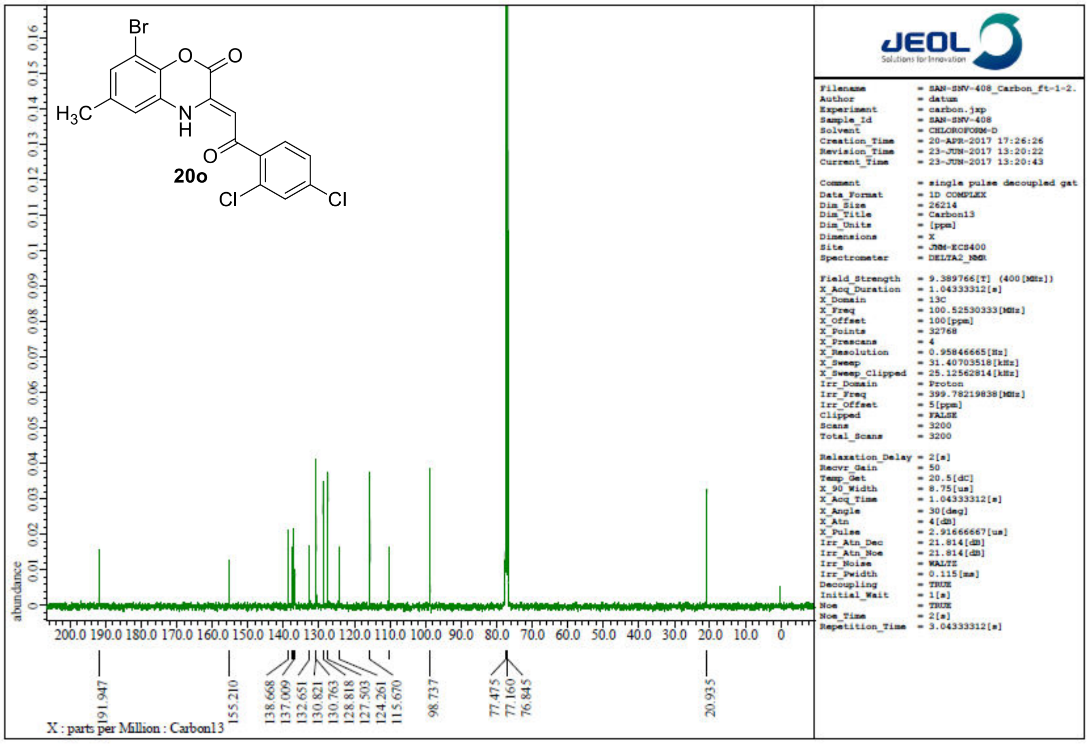

Figure 14. ^{13}C NMR Spectra of Compound **20o**.

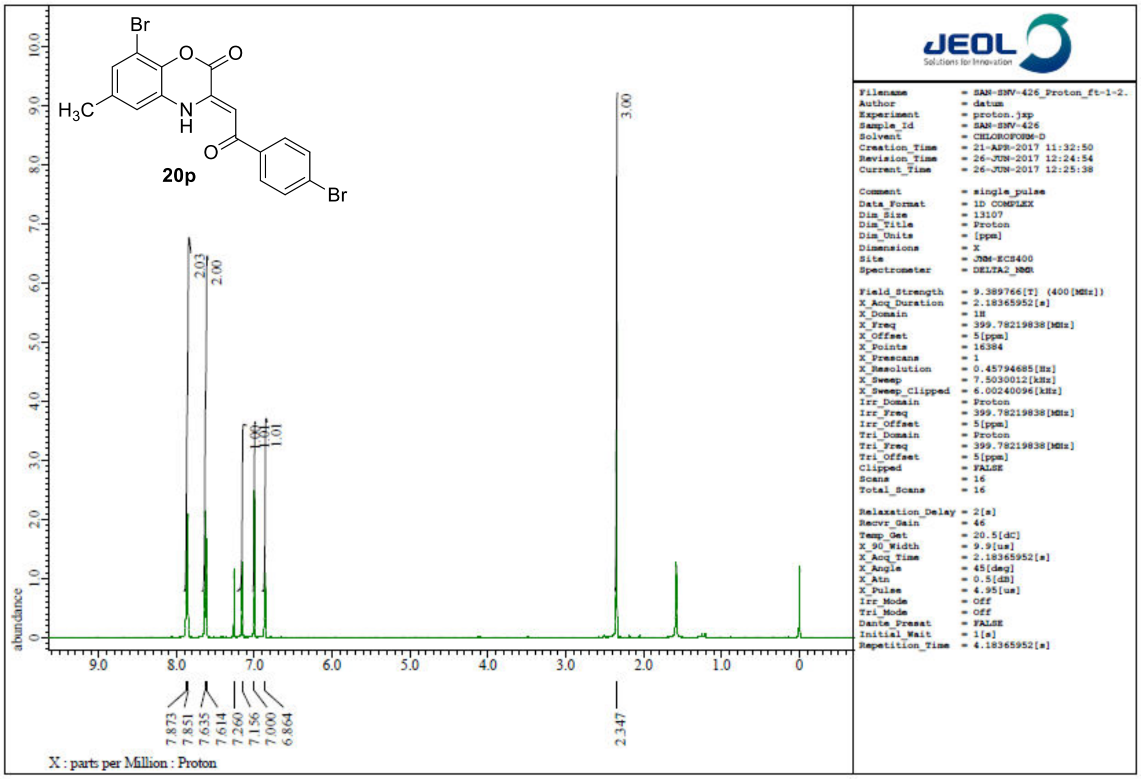

Figure 15. ^{1}H NMR Spectra of Compound **20p**.

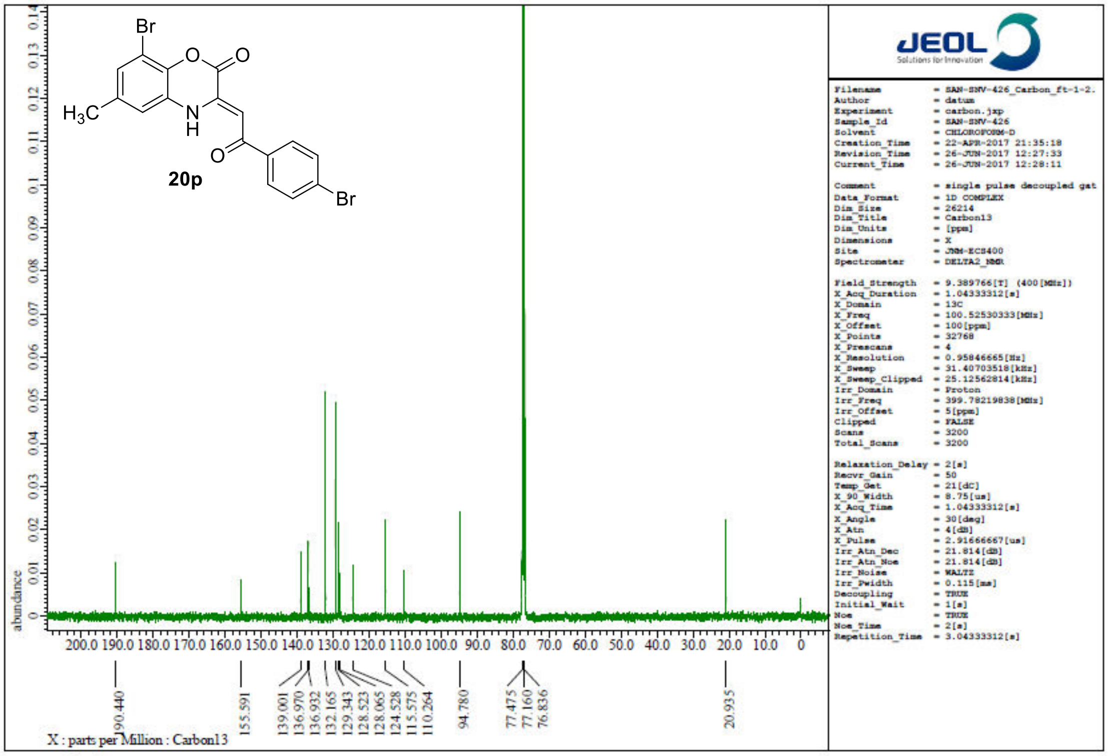

Figure 16. ^{13}C NMR Spectra of Compound **20p**.

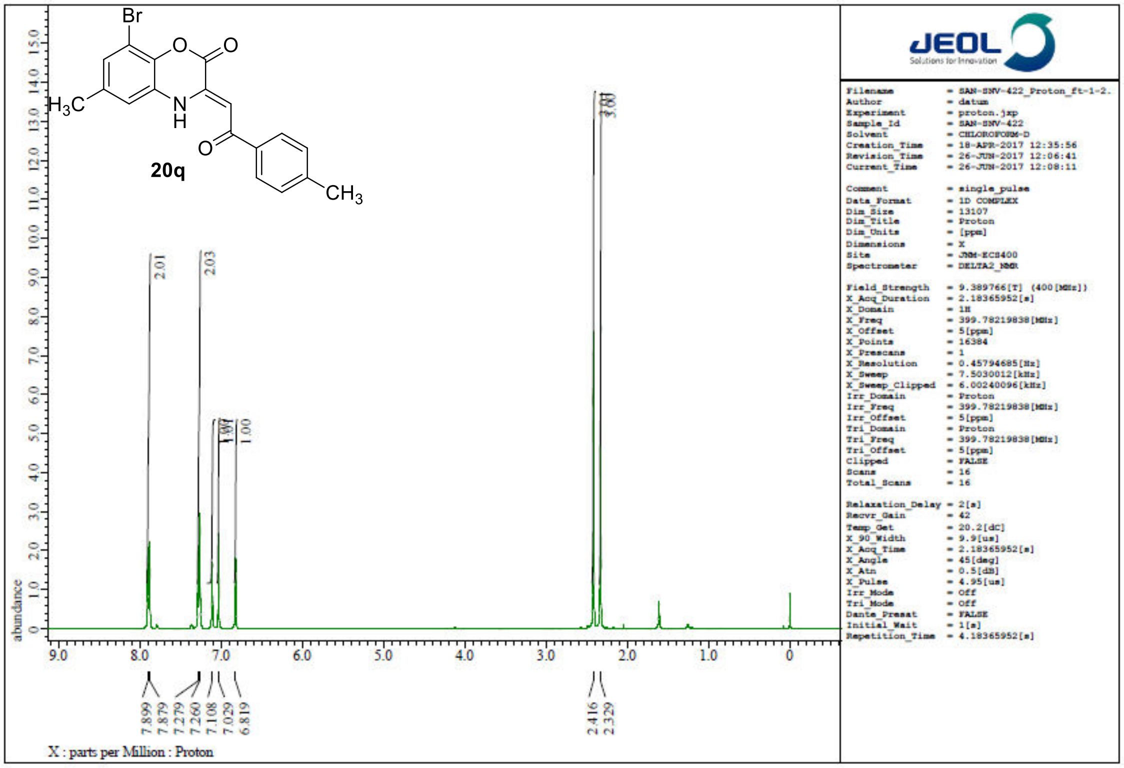

Figure 17. ^{1}H NMR Spectra of Compound **20q**.

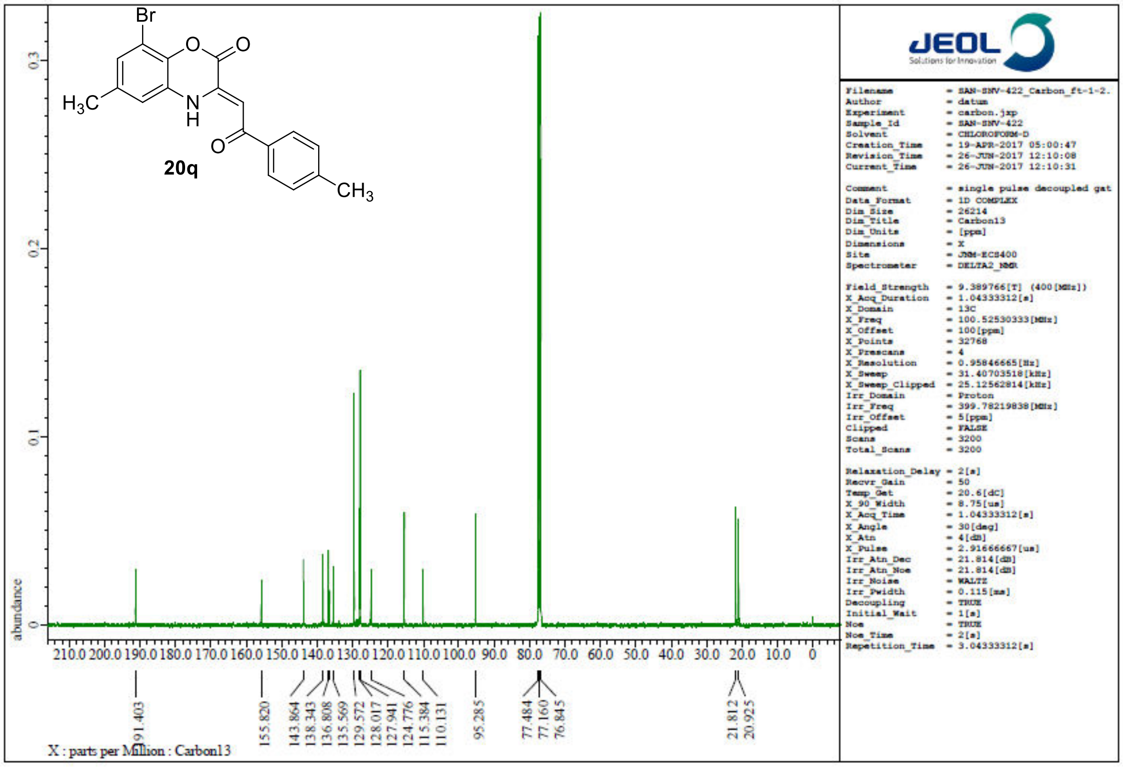

Figure 18. ^{13}C NMR Spectra of Compound **20q**.

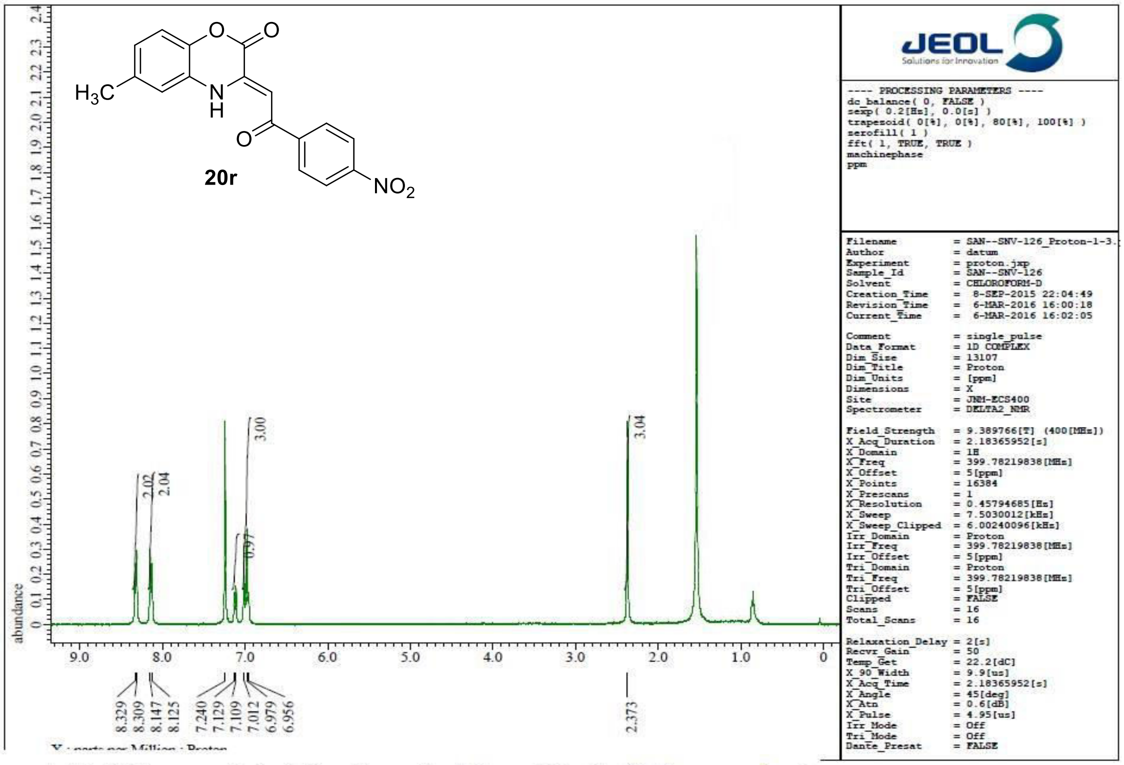

Figure 19. ^{1}H NMR Spectra of Compound **20r**.

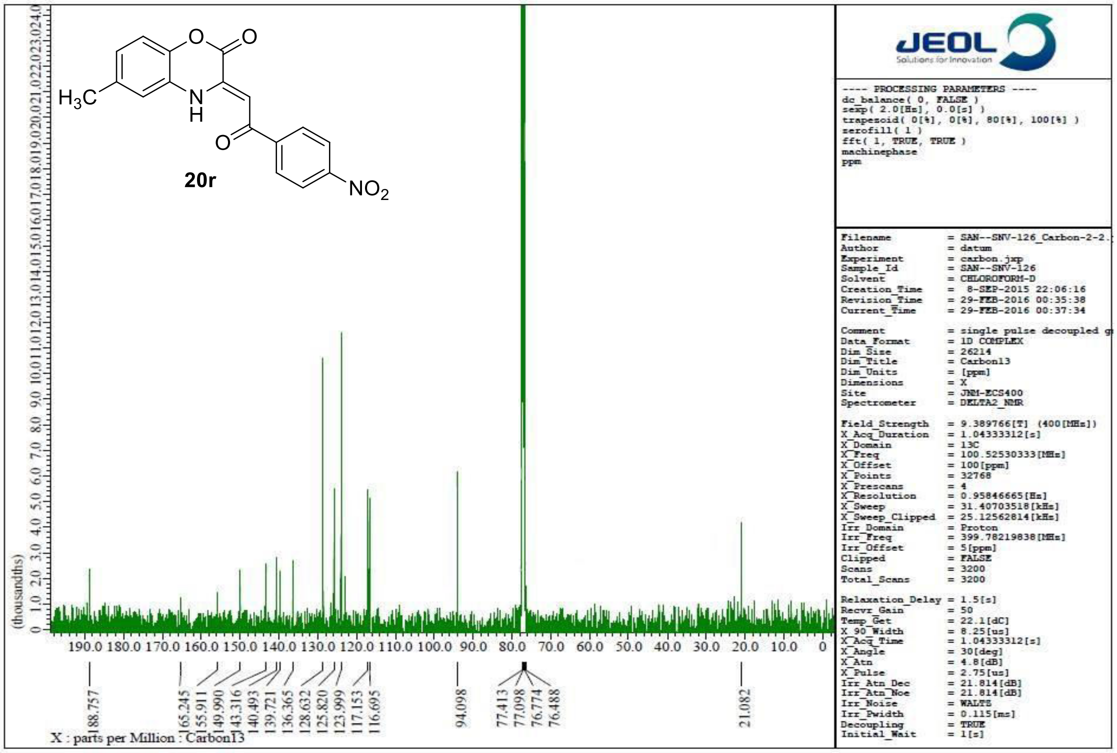

Figure 20. ^{13}C NMR Spectra of Compound **20r**.

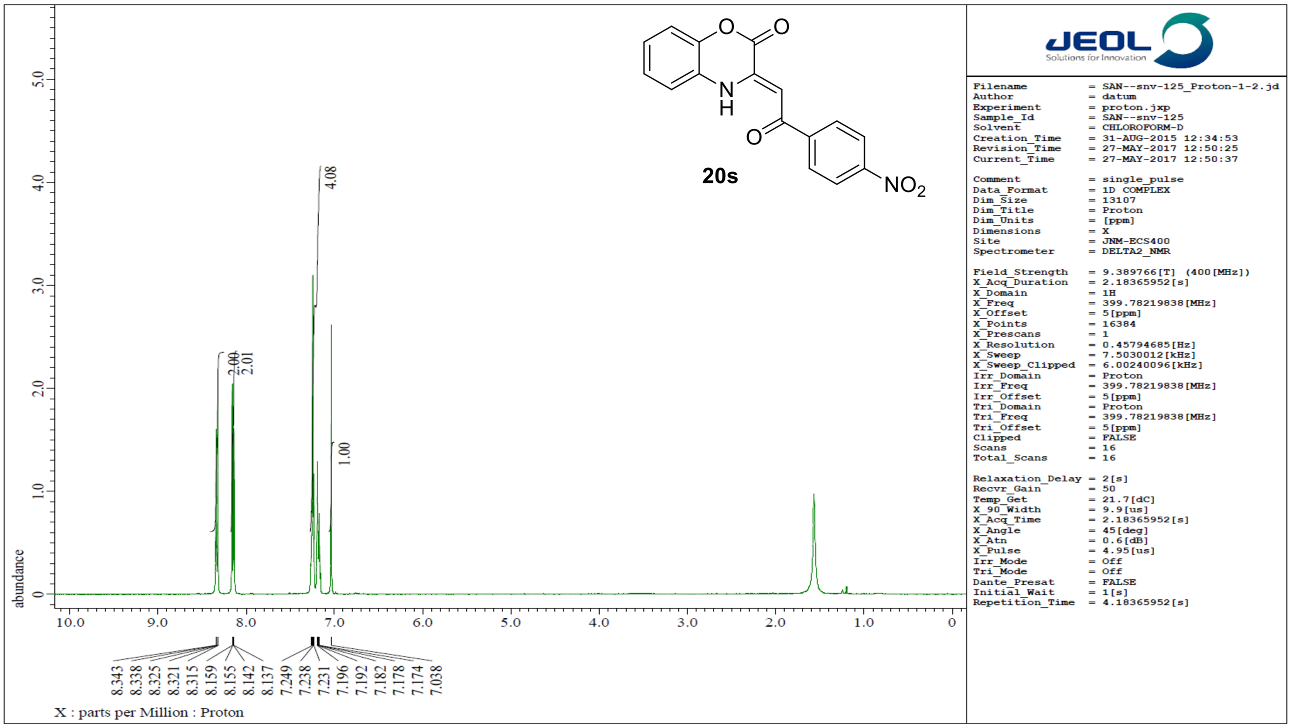

Figure 21. ^{1}H NMR Spectra of Compound **20s**.

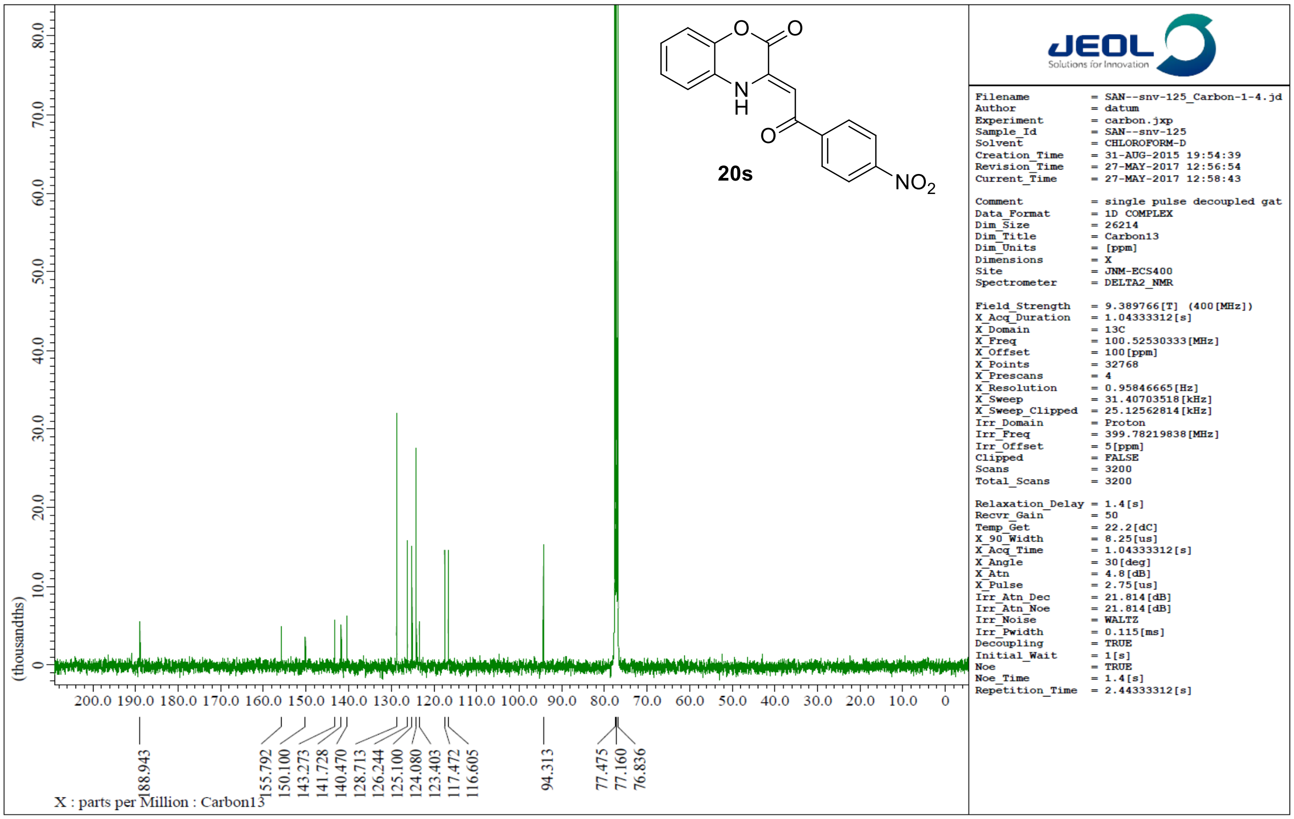

Figure 22. ^{13}C NMR Spectra of Compound **20s**.

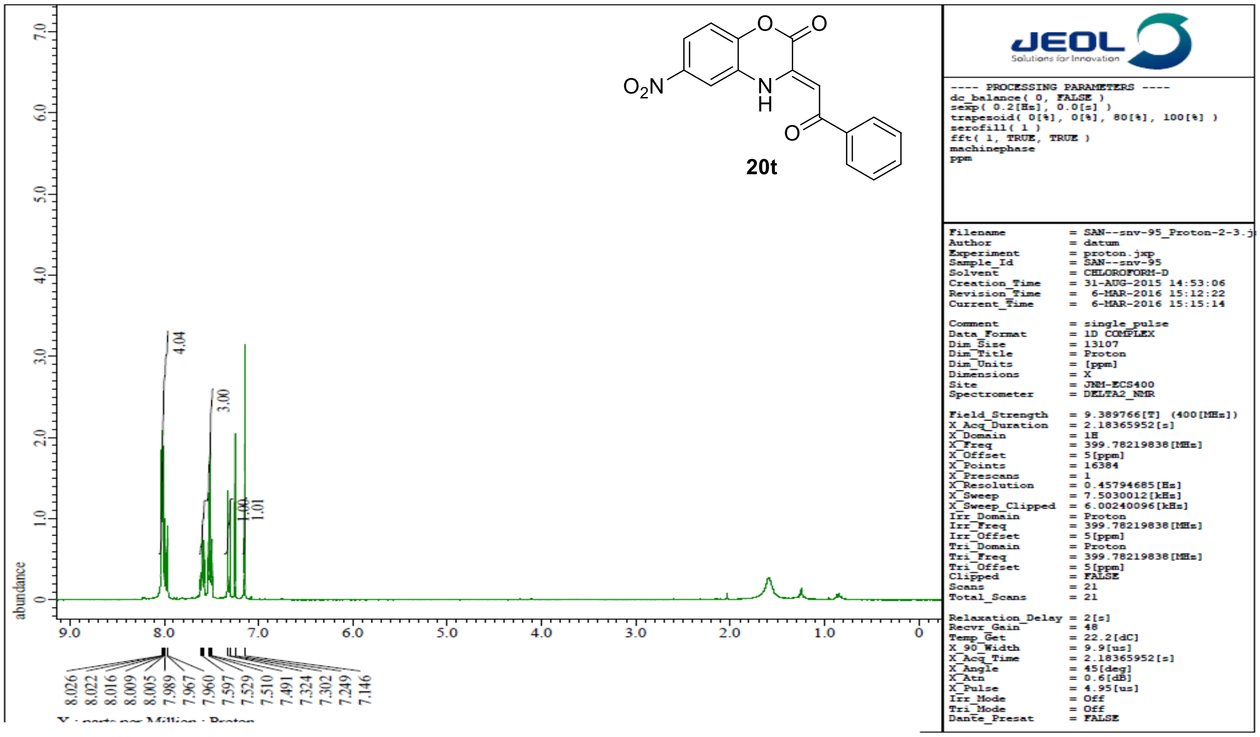

ou created this PDF from an application that is not licensed to print to novaPDF printer (http://www.novapdf.com)

Figure 23. ^{1}H NMR Spectra of Compound **20t**.

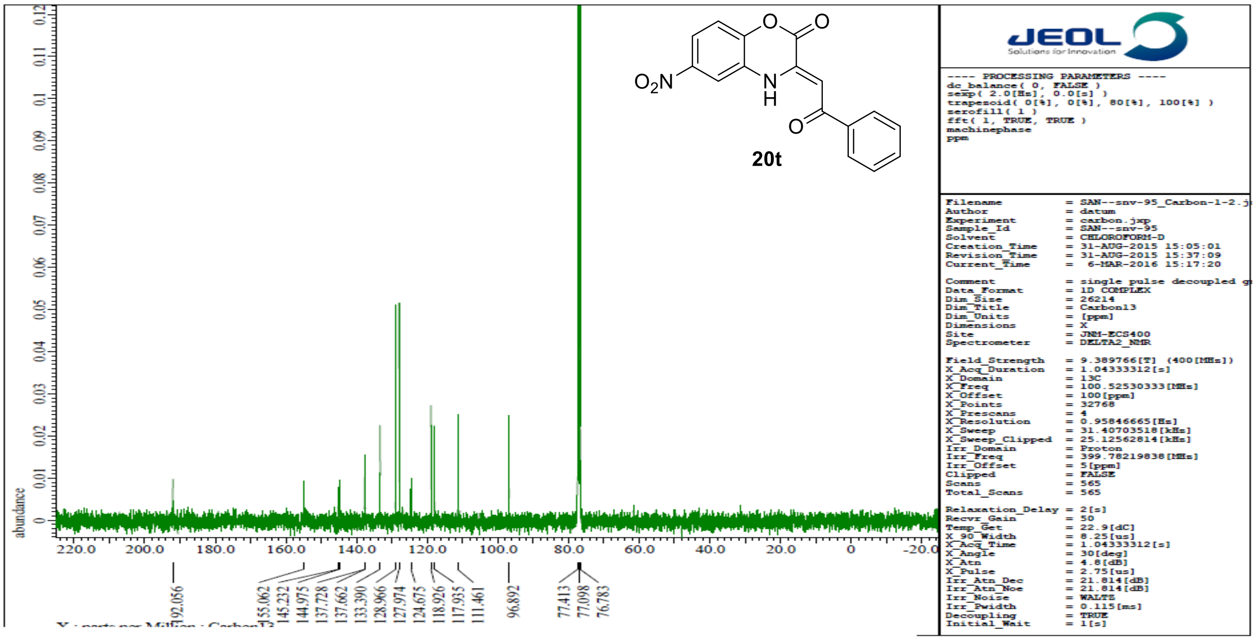

ou created this PDF from an application that is not licensed to print to novaPDF printer (http://www.novapdf.com)

Figure 24. ^{13}C NMR Spectra of Compound **20t**.

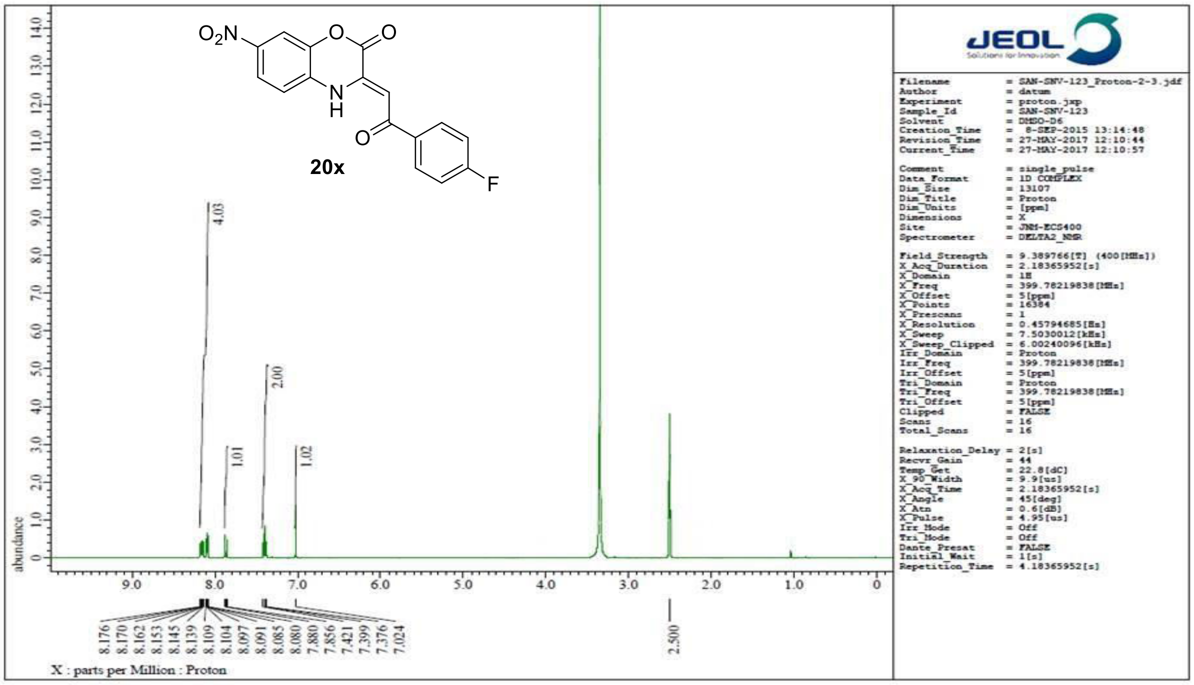

ou created this PDF from an application that is not licensed to print to novaPDF printer (http://www.novapdf.com)

Figure 25. ^{1}H NMR Spectra of Compound **20x**.

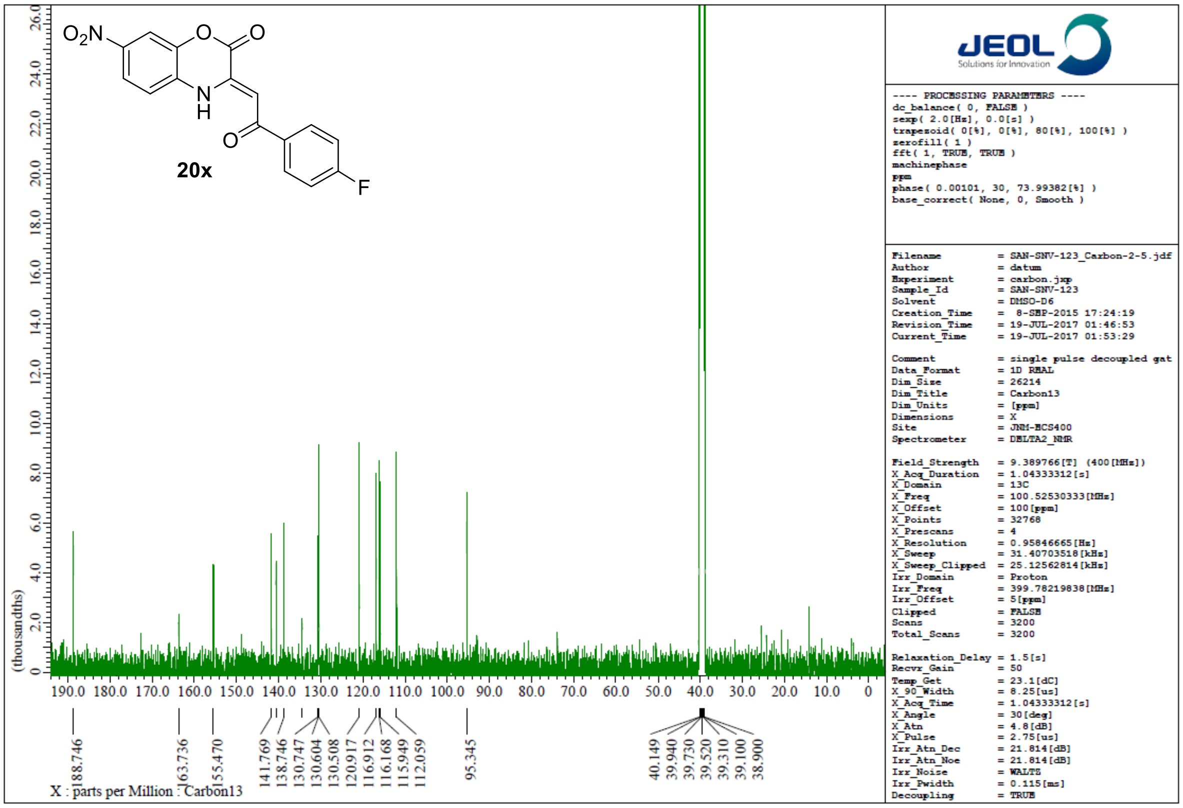

Figure 26. ^{13}C NMR Spectra of Compound **20x**.

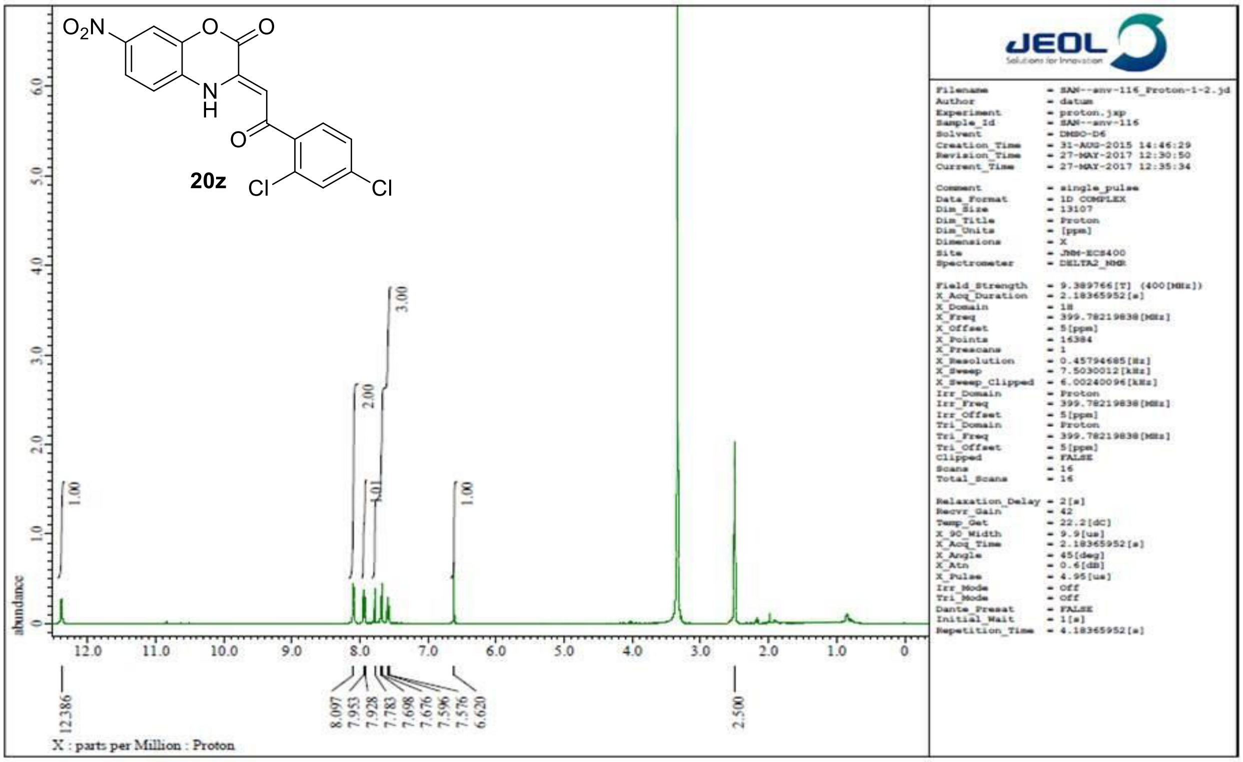

ou created this PDF from an application that is not licensed to print to novaPDF printer (http://www.novapdf.com)

Figure 27. ^{1}H NMR Spectra of Compound **20z**.

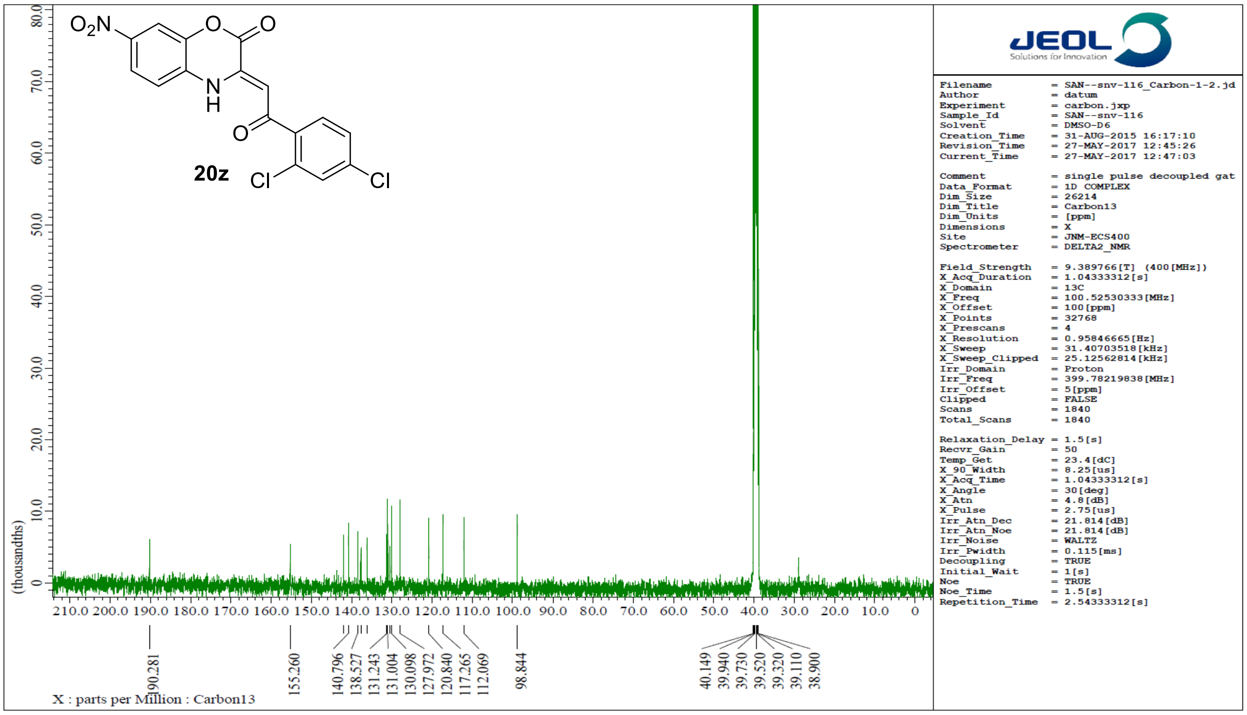

Figure 28. ^{13}C NMR Spectra of Compound **20z**.

Cytotoxicity Assay

The cytotoxic effect of the active compounds prepared on cells was detected *in vitro* using the mitochondrial cytotoxic test according to Danihelová et al. (2013) with modifications. Cell viability was evaluated using thiazolyl blue tetrazolium bromide (MTT), which indicates the metabolic activity of cells. The experiment was performed in 96-well microplates. The cells were seeded at a density of 3.5×10^3 3T3 fibroblast cells per well. Samples were dissolved in DMSO (stock solution 10 mM) and subsequently diluted in medium to the final concentration of 25 µM to 250 µM (concentration of DMSO 0.5%) and after 24 h they were added to the cells. Microplates were cultivated for 72 h in thermostat at 37 °C and 5% CO_2 atmosphere. After incubation thiazolyl blue tetrazolium bromide (3.33 mg/ml phosphate buffered saline, pH=7.4) was pipetted to each well and left to incubate for further two hours. Then the medium with MTT solution was removed. Formazan crystals in viable cells were dissolved in the lysis solution (4 mM HCl and 0.1% Nonidet P40 in ethanol). Microplates were shaken 15 min at 1500 rpm. Absorbance was measured at 540 nm and reference wavelength at 740 nm. Each value is the mean of 6 wells with standard deviation. Inhibition activity was expressed as percentages of control with DMSO.

frontiers
